# Uncovering the Prevalence and Diversity of Integrating Conjugative Elements in Actinobacteria

**DOI:** 10.1371/journal.pone.0027846

**Published:** 2011-11-16

**Authors:** Mariana Gabriela Ghinet, Eric Bordeleau, Julie Beaudin, Ryszard Brzezinski, Sébastien Roy, Vincent Burrus

**Affiliations:** Centre d'étude et de valorisation de la diversité microbienne, Département de biologie, Université de Sherbrooke, Sherbrooke, Québec, Canada; University of Hyderabad, India

## Abstract

Horizontal gene transfer greatly facilitates rapid genetic adaptation of bacteria to shifts in environmental conditions and colonization of new niches by allowing one-step acquisition of novel functions. Conjugation is a major mechanism of horizontal gene transfer mediated by conjugative plasmids and integrating conjugative elements (ICEs). While in most bacterial conjugative systems DNA translocation requires the assembly of a complex type IV secretion system (T4SS), in Actinobacteria a single DNA FtsK/SpoIIIE-like translocation protein is required. To date, the role and diversity of ICEs in Actinobacteria have received little attention. Putative ICEs were searched for in 275 genomes of Actinobacteria using HMM-profiles of proteins involved in ICE maintenance and transfer. These exhaustive analyses revealed 144 putative FtsK/SpoIIIE-type ICEs and 17 putative T4SS-type ICEs. Grouping of the ICEs based on the phylogenetic analyses of maintenance and transfer proteins revealed extensive exchanges between different sub-families of ICEs. 17 ICEs were found in Actinobacteria from the genus *Frankia*, globally important nitrogen-fixing microorganisms that establish root nodule symbioses with actinorhizal plants. Structural analysis of ICEs from *Frankia* revealed their unexpected diversity and a vast array of predicted adaptive functions. *Frankia* ICEs were found to excise by site-specific recombination from their host's chromosome *in vitro* and *in planta* suggesting that they are functional mobile elements whether Frankiae live as soil saprophytes or plant endosymbionts. Phylogenetic analyses of proteins involved in ICEs maintenance and transfer suggests that active exchange between ICEs cargo-borne and chromosomal genes took place within the Actinomycetales order. Functionality of *Frankia* ICEs *in vitro* as well as *in planta* lets us anticipate that conjugation and ICEs could allow the development of genetic manipulation tools for this challenging microorganism and for many other Actinobacteria.

## Introduction

Actinobacteria are found in many different ecological niches. These high G+C Gram-positive bacteria are soil and aquatic inhabitants (e.g., *Streptomyces*, *Micromonospora*, *Rhodococcus*), plant symbionts (e.g., *Frankia*), plant and animal pathogens (e.g., *Corynebacterium*, *Mycobacterium*, *Nocardia*), or gastrointestinal commensals (e.g., *Bifidobacterium*). Nitrogen-fixing actinobacteria belonging to genus *Frankia* live as soil saprophytes and as endophytic symbionts in over 200 plant species [1]. These host plants (actinorhizal plants) are found on nearly all continents, and the contribution of *Frankia* and actinorhizal plants to global nitrogen fixation is estimated at 25% [2], [3]. Frankiae are understudied compared to other actinomycetes and this is largely attributable to difficulties in isolation and maintenance of actively proliferating cultures. Nevertheless, the global environmental importance of Frankiae command more research to better understand their interactions with other microorganisms, and host plants.

Frankiae have circular chromosomes, in contrast to streptomycetes. Genome sizes are highly variable, as exemplified by strains Cci3, ACN14a and EAN1pec (5.4, 7.5, and 9.4 Mbp, respectively) [4]. This diversity suggests the existence of a spectrum of lifestyles ranging from quasi-obligatory symbiosis (small genome size) to free-living status with the possibility of symbiosis in a wide array of host plants (large genome size). The size of the larger genomes of Frankiae is also reminiscent of that of related, GC-rich, biotechnologically important genus *Streptomyces.* However, in great contrast to what is known of streptomycete genetics, much remains to be learned about the horizontal exchange of genetic material in Frankiae. Such knowledge is essential to decipher the evolution of members of this genus and to achieve stable genetic manipulation in these strains – a goal that has eluded researchers to date.

A growing number of reports indicate that horizontal gene transfer is an essential mechanism by which some actinobacterial species acquired pathogenesis-related functions [5], [6], [7]. Mobile genetic elements are also responsible for extensive genomic rearrangements revealed by structural genomics and observed even among related actinobacterial species [8]. Actinomycete integrative and conjugative elements (AICEs), *aka.* integrating conjugative plasmids, are particularly prevalent in the genomes of several species of *Streptomyces* [9]. AICEs belong to the broader class of integrative and conjugative elements (ICEs) usually described in Gram-negative bacteria and in the Firmicutes. Like temperate bacteriophages, ICEs integrate into and replicate with the chromosome of the bacterial host [10], [11], [12]. The site-specific integration of ICEs in the host chromosome is usually mediated by a tyrosine recombinase or occasionally by a serine recombinase, encoded by an *int* gene. Site-specific recombination occurs between two short identical or nearly identical sequences located in attachment sites, one on the element (*attP*) and the other one on the chromosome (*attB*). Upon various conditions, ICEs can excise from the chromosome to form circular covalently closed molecules. Like most conjugative plasmids, ICEs disseminate via conjugation. In Gram-negative bacteria and Firmicutes, conjugative DNA transfer typically requires the assembly of a type IV secretion system (T4SS) which is involved in translocation of the DNA to recipient cells [13]. Biochemical processing of the DNA molecule to transfer is initiated at a specific *cis*-acting site called the origin of transfer (*oriT*), which is bound by a DNA relaxase (Mob protein) and other auxiliary proteins, forming altogether a nucleoprotein complex called the relaxosome [14]. The phosphodiesterase activity of the relaxase mediates a strand-specific cleavage within *oriT*, allowing the unwinding and 5′ to 3′ transfer of a single-stranded DNA (T-strand) to the recipient cell. A coupling protein (T4CP) links the relaxosome to the T4SS and is thought to form the conjugative pore through which the T-strand is translocated to the recipient cell. While in the donor cell the complementary strand is used as a template for replacement strand synthesis, within the recipient cell host the T-strand is converted into double-stranded DNA that can be either re-circularized and/or recombined into the recipient chromosome.

Interestingly, inter-mycelial conjugative transfer of ICEs identified in actinomycetes significantly differs from this general mechanism as it proceeds via translocation of unprocessed double-stranded DNA. This mechanism is reminiscent of chromosome partitioning during sporulation and cell division (reviewed by te Poele et al. [9]). Furthermore, once excised from the chromosome, actinomycete ICEs replicate autonomously like genuine plasmids [15], [16]. They encode a replication initiator protein and carry an origin of replication. Mutations in either of these two elements has been shown to lead to loss of transfer of the prototypical actinomycete ICE pSAM2, indicating that autonomous replication is required for conjugative transfer to recipient cells [17], [18], [19]. AICEs have been developed into useful tools for genetic engineering of actinobacteria [20].

In this study, we investigated the prevalence and diversity of ICEs in Actinobacteria, and focused particularly on ICEs in six *Frankia* strains, EAN1pec, ACN14a, Cci3, EuI1c, EUN1f and *Frankia* symbiont of *Datisca glomerata*. Prior to this study, three AICEs, Fean5323, Fean6303 and Faln5456 originating from two members of the genus *Frankia*, have been briefly described [9]. Our study reports the frequent occurrence of AICEs in *Frankia* genomes. Their ability to excise from the chromosome as circular molecules both *in vitro* and *in planta* was examined as excision is the initial step preceding their eventual transfer to a recipient cell via conjugation.

## Results and discussion

### Identification of putative AICEs in the genomes of Actinobacteria

**Figure 1:**
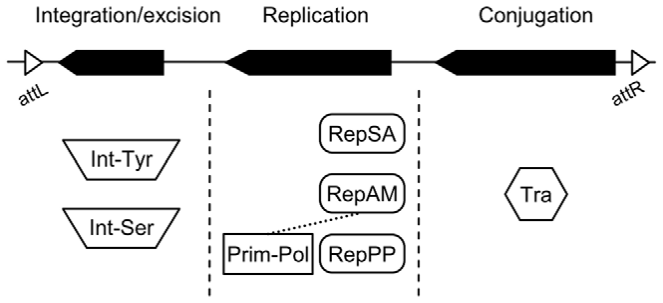
Schematic representation of canonical AICEs modules and corresponding protein combinations. Possible combination of integration/excision, replication and conjugative transfer modules are represented. For each functional module, specific domain-containing proteins where searched for as follow: Tra (FtsK/SpoIIIE, PF01580), Int-Tyr (Phage integrase, PF00589), Int-Ser (Recombinase, PF07508), RepSA (this study), RepAM (DUF3631, PF12307) and Prim-pol (Prim-pol, PF09250). AICEs Prim-pol proteins are encoded along with putative replication proteins (RepPP) or can be associated with RepAM replication initiator proteins.

To address the prevalence, repartition and diversity of AICEs in Actinobacteria, we carried out a large-scale *in silico* analysis using data extracted from the RefSeq database [21]. The predicted proteomes encoded by 275 genomes of Actinobacteria were screened using HHM profiles of protein orthologs associated with the functions of core modules of ICEs previously identified in actinomycetes [9] (Figure 1 and Table 1): (i) integrases (Int) of the tyrosine or serine family of recombinases that are involved in ICE integration and excision, (ii) replication initiator proteins (Rep) of the RepSA- and RepAM-type, and putative polymerases of the Prim-Pol-type (bifunctional DNA primase/polymerase), and (iii) FtsK/SpoIIIE domain-containing proteins (Tra) that are involved in translocation of double-stranded chromosomal DNA. This analysis revealed a large number of possible AICE-associated proteins (Table 2); yet many of these hits may be part of other types of mobile elements (transposons, prophages, integrated plasmids) or have functions unrelated to mobile elements. For instance, FtsK/SpoIIIE domains are known to be found in proteins rescuing the remainder of chromosomal DNA into the appropriate daughter-cell compartments during constriction of the septal membranes in prokaryotic cell division (FtsK) [22], [23], [24] and in asymmetric division of sporulating *Bacillus subtilis* (SpoIIIE) [25]. Similarly, subsets of tyrosine recombinases such as MrpA encoded by the *Streptomyces coelicolor* A3(2) plasmid SCP2 [26] catalyze the resolution of plasmid multimers into the monomeric form after replication. Therefore, to limit our investigation to genuine putative AICEs, 30- to 60-kb chromosomal regions containing genes encoding at least one of each of the three components (Int, Tra and Rep) were further considered in our analyses (Figure 1). However, we excluded the very few cases for which the *int* gene was located in between the *rep* and *tra* genes as recombinase genes are typically located adjacent to one of the two *att* sites flanking an integrated ICE or prophage. The chromosomal fragments coding for two or less of these components were also excluded and likely correspond to AICE remnants, prophages, transposons and other genomic islands. A total of 139 putative AICEs were identified in chromosomal sequences (Table 2 and S1). In addition, the analysis of 176 actinobacterial plasmids revealed 4 additional ICEs: the mega plasmid pNDAS01 contains a putative ICE, while plasmids pSA1.1, pSLS and pWTY27 likely correspond to ICEs in their excised form (Table S1).

**Table 1:** Pfam HMM profiles used in this study.

Function	Domain name	Accession number	Description
**Integration**	Phage_integrase	PF00589	Tyrosine recombinase
	Recombinase	PF07508	Serine recombinase
**Replication**	RepSA	This study	Replication initiator protein, pSAM2
	RepAM, DUF3631	PF12307	Replication initiator protein, pMEA300
	Prim-Pol	PF09250	Bifunctional DNA primase/polymerase, N-terminal
	Rep_1	PF01446	RCR replication protein
	Rep_2	PF01719	Plasmid replication protein
	Rep_3	PF01051	Initiator of plasmid replication
	RepA_C	PF04796	Plasmid encoded RepA protein
	Replicase	PF03090	Replication initiator protein
	Rep_trans	PF02486	Replication initiation factor
	Phage_rep_O	PF04492	Bacteriophage replication protein O
	RepL	PF05732	Firmicute plasmid replication protein
	Phage_CRI	PF05144	Phage replication protein CRI
	Phage_GPA	PF05840	Bacteriophage replication gene A protein
	IncFII_repA	PF02387	IncFII RepA protein
	RP-C	PF03428	Viral replication protein C, N-terminal domain
	RepA_N	PF06970	Replication initiator protein A (RepA), N-terminus
	RepC	PF06504	Replication protein C
	RPA	PF10134	Replication initiator protein A
**DNA transfer**	FtsK_SpoIIIE	PF01580	Intercellular chromosomal DNA transfer
	T4SS-DNA_transf	PF02534	Coupling protein (VirD4-like T4CP)
	AAA_10	PF12846	ATPases Associated with diverse cellular Activities (VirB4-like T4SS component)
	TrwC	PF08751	TrwC relaxase

**Table 2:** Pfam HMM profiles used in this study.

	Number of hits	
Domains	complete^a^	draft^b^
Tyrosine integrase	1029	931
Serine integrase	143	139
FtsK/SpoIIIE	882	1012
RepSA-like	76	64
RepAM (DUF3631)	24	69
Prim-Pol	99	179
Rep2	7	6
**AICEs**	73	67

*^a^*130 complete genomes

*^b^*145 draft genomes

**Figure 2:**
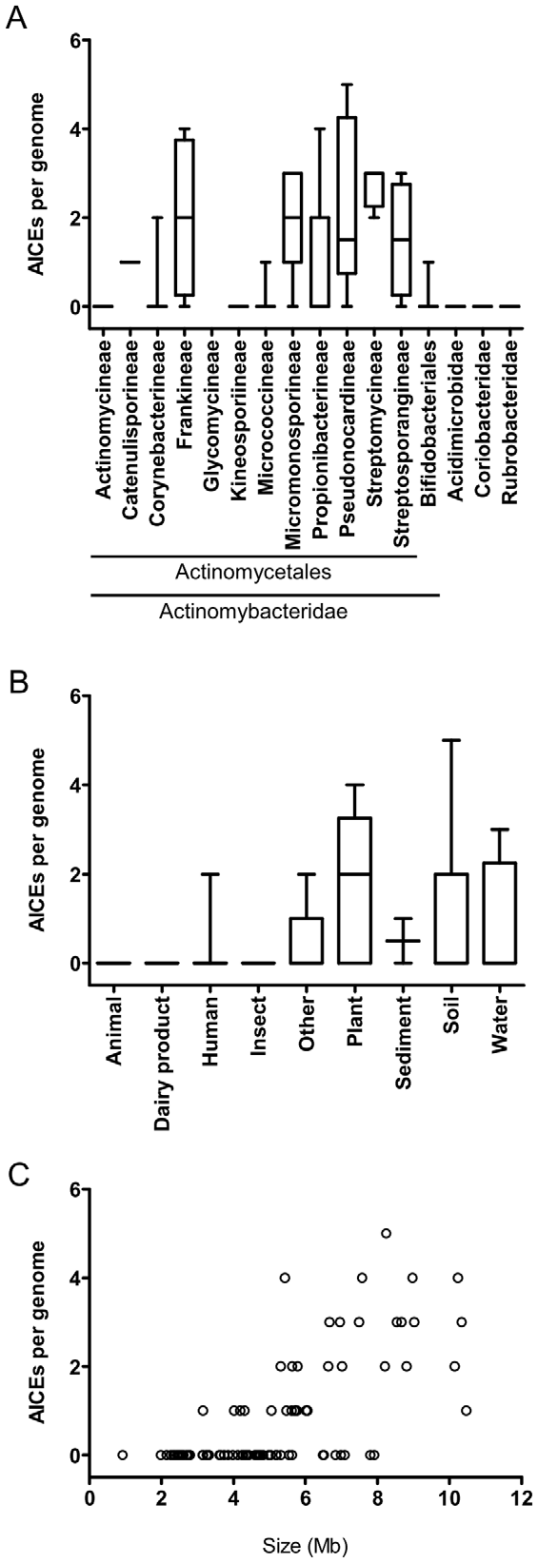
Distribution of AICEs in completed genomes. (A) Distribution per suborder of actinobacteria. (B) Distribution per environmental niche of the original host. (C) Distribution as a function of genome size. (A and B) Median, standard deviation (rectangles) and extreme values (brackets) are shown. (B and C) Only the *Actinomycetales* are shown.

This first *in silico* analysis unambiguously showed that based on the criteria indicated above, AICEs are constrained to members of the *Actinomycetales* order (Figure 2A). On the contrary, the closely related *Bifidobateriales* order, and the other Actinobacteria subclasses (*Acidimicrobidae*, *Coriobacteridae* and *Rubrobacteridae*) appeared to be completely devoid of AICEs. This observation echoes a similar conclusion that was drawn by te Poele et al. [9] from a smaller genome sample. However, since this initial analysis was based on specific assumptions concerning the nature and gene conservation among known AICEs, it was inherently biased. To circumvent this bias, we extended our analysis to include 15 other families of replication initiator proteins of plasmid or viral origin. Surprisingly, using this approach, we were able to identify only one additional putative ICE, which relies on a Rep2-type replication initiator protein, in a strain of *Bifidobacterium longum* (Table S1).

We then analyzed the distribution of AICEs among Actinobacteria as a function of their host's environmental niche. In general, species isolated from plant, soil and water samples tend to contain a significantly larger number of AICEs per genome than those isolated from dairy products, animal, insects or sediments (Figure 2B). This observation suggests that AICEs could confer specific advantages to their respective bacterial hosts in these specialized ecological niches. However, additional extensive sequence analyses and functional characterization of the gene cargo borne by AICEs will be necessary to determine conclusively what benefits these mobile elements confer to their host. As expected, large actinomycete genomes tend to bear significantly more AICEs than small genomes (Figure 2C). It is not clear to date whether the acquisition of multiple AICEs by a single genome is constrained by specific barriers, such as the impossibility for two different AICEs to occupy the same integration site and form tandem arrays, or by the existence of specific AICE-encoded immunity- or exclusion-like systems. For example, Pif, a NUDIX hydrolase domain-containing protein encoded by pSAM2, has been shown to act in the recipient cell to prevent redundant exchange between two cells harboring the same conjugative element [27]. According to our analysis, AICEs often bear genes encoding NUDIX hydrolase domain-containing proteins, suggesting that conjugal immunity is widespread (data not shown). However, the molecular mechanism of conjugal immunity mediated by proteins such as Pif and the range of AICEs that a single Pif-like protein can target for immunity remain unknown.

### AICE recombination functions and integration sites

**Figure 3:**
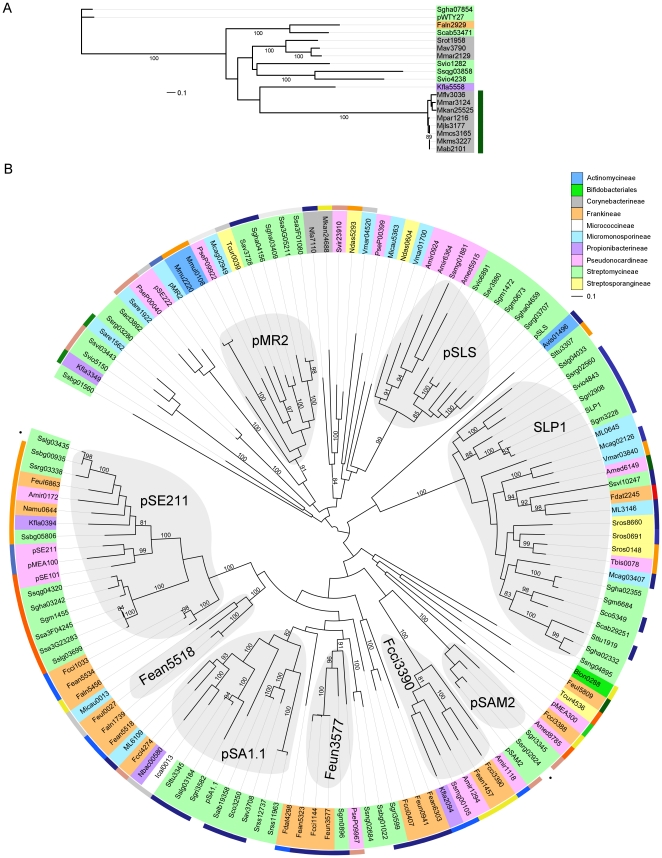
Phylogenetic trees of the site-specific recombinases found in AICEs. (A) Serine recombinases. (B) Tyrosine recombinases. Sub-families were defined as clades containing at least 4 members with a distance cut-off of 1 and with a >80 bootstraps support. Label colors indicate the suborders of Actinomycetales within which the AICEs were found (see legend). Colors in the outer wheel in panel A and the dark green strip in panel B indicate the type of tRNA genes into which each AICE is integrated (see Table S1 for details). Black dots in panel A for Ssrg02924 and Sslg03435 indicate that while no tRNA-encoding gene was detected as their integration sites, the *att* sites nearest to their respective *int* genes were identical to the ones of their closest relatives, i.e. pSAM2 and Ssbg00935, respectively.

Among the 144 AICEs that we detected in our analysis, only 19 (∼14%) were found to rely on an integrase of the serine recombinase family for their integration into and excision from the chromosome (Figure 3A and Table S1). All the other AICEs appear to rely on integrases of the tyrosine recombinase family instead (Figure 3B and Table S1). This imbalanced proportion correlates rather well with what is generally observed for ICEs and temperate bacteriophages; yet surprisingly, more than half of the AICEs encoding serine recombinases were found in the order *Corynebacterineae*. In fact, all *Mycobacterium* AICEs but Mkan24688 encode serine recombinases, which clustered in two groups. The first group contains the recombinases encoded by Mmar2129 and Mav3790 as well as Srot1958 from *Segniliparus rotundus*, another member of *Corynebacterineae*. These AICEs are integrated into protein-encoding genes. The second group has 8 recombinases which originate from different species of *Mycobacterium* yet exhibit high sequence similarities, and catalyze the integration into the same tRNA Leu gene (Figure 3A). Serine recombinase-mediated integration into tRNA genes is rare [28]. In fact to our knowledge, only ϕRSM1, a filamentous phage infecting *Ralstonia solanacearum*, was found to encode a serine recombinase catalyzing its integration into the 3′ end of a tRNA Ser gene [29]. Interestingly, no AICE was detected in any of the 30 genomes of *Mycobacterium tuberculosis* or in the 3 genomes of *Mycobacterium bovis* that were part of our analysis.

Phylogenetic analyses of 125 AICE integrases of the tyrosine recombinase family revealed several groups of proteins clustering into subfamilies which possess more extensive amino acid sequence similarities (Figure 3B). Eight such groups containing four or more integrases were identified in the phylogenic tree of tyrosine recombinases. One of these subfamilies, pSE211, was previously identified by Williams [28]. Eight new subfamilies clearly emerge from our analysis, SLP1, pSLS, pMR2, pSAM2, Fcci3390, pSA1.1, Feun3577 and Fean5518. No single subfamily was found to be exclusive of a particular species, genus or even sub-order. One of the possible benefits for AICEs of the availability of such large pool of different integrase subfamilies is that they are more likely to target different integration sites, allowing them to stably coexist within the same genome. Different mobile genetic elements sharing the same integrase and integration site have been shown to form tandem arrays that are inherently unstable, leading to recombination between elements composing the array, incompatibility and/or loss [30], [31], [32], [33], [34].

We observed that the vast majority of AICE encoding tyrosine recombinases were integrated in the 3′ end of a tRNA gene, with the exception of all AICEs encoding integrases belonging to the pSLS sub-family. Several past reports revealed a strong preference for tRNA and tmRNA gene sequences as prophage integration sites [28], [35], [36]. In at least two instances, i.e. Sslg03435 and Ssrg2924, no tRNA gene could be readily identified as the integration site by tRNAscan-SE; yet the *att* sequences flanking these two AICEs were found to be identical to the *att* sites flanking Ssbg03435 and pSAM2 respectively, which encode closely related integrases and integrate into the 3′ end of tRNA genes (Figure 3B). This observation indicates that probable tRNA gene remnants can be suitable integration sites. As expected, closely related integrases seem to target the same tRNA gene in their respective hosts. For example, the 6 closely related pSE211-type integrases encoded by the group Ssqg04320/Sslg03699 catalyze integration into a tRNA-Thr gene. Closely related integrases can also catalyze integration into different tRNA genes, as observed for Fcci1033/Fean5534 (tRNA-Lys) and Faln5456 (tRNA-Met). Mobile elements such as prophages and ICEs likely take advantage of the frequent association between tyrosine recombinase with tRNA genes as integration sites. While the benefits of such associations have not yet been fully elucidated, several suitable hypotheses have been proposed including sequence reliability over protein-coding sequences, transcriptional coupling and so on [28].

### Replication functions of AICEs

**Figure 4:**
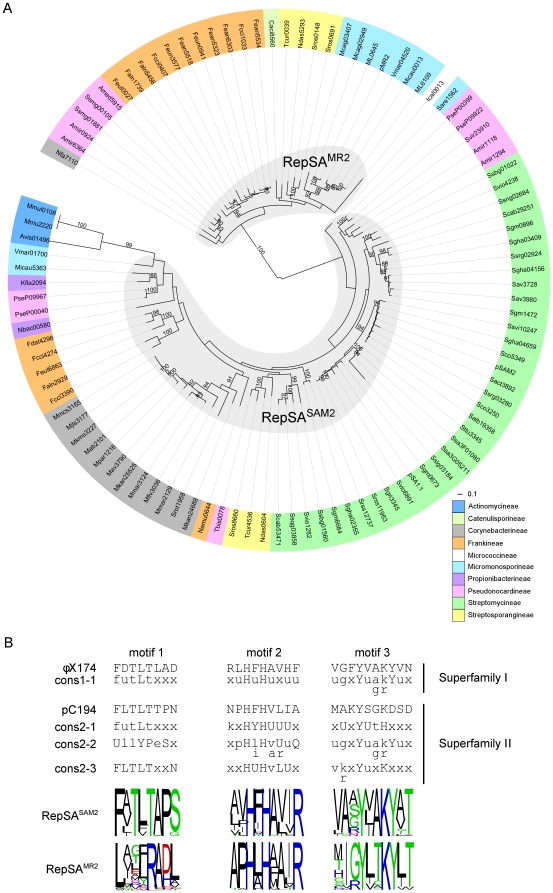
Evolutionary relationship of RepSA-like rolling-circle initiator proteins found in AICEs. (A) Phylogenetic tree of RepSA proteins. Sub-families were defined as clades containing at least 4 members with a distance cut-off of 1 and with a >80 bootstraps support. Label colors indicate the suborders of Actinomycetales within which the AICEs were found (see legend). (B) Comparison of the conserved motifs found in RepSA-like proteins from AICEs with the conserved motifs found in the catalytic domain of rolling-circle initiator proteins of plasmids and single-stranded DNA coliphages. The consensus sequences shown are as previously reported by Del Solar et al. [37]. Motif 2 (HUH or His-hydrophobic-His) corresponds to the putative divalent cation-binding site and motif 3 contains the tyrosine catalytic residue. Mmu2220, Mmul0108 and Avis01496 were not included in the alignment used to generate the logo sequence of the RepSA^SAM2^ conserved motifs.

Replication of the first described AICE, pSAM2, was found to be dependent upon the protein RepSA [17], [18]. As other replication initiator proteins involved in rolling-circle replication (RCR), RepSA was shown to bind to the *binding region* of the *double-stranded origin* (*dso*) and to introduce a site-specific nick in the *nick region* of *dso*, leaving a 3′-OH end used for priming the elongation by host encoded replication proteins (DNA polymerase III, helicase) [37]. Surprisingly, phylogenetic analysis of replication initiator proteins detected with the RepSA HMM profile that we generated, revealed clustering in two major distantly related subfamilies that were not previously reported as phylogenetically distinct by te Poele et al. [9]. The first subfamily, RepSA^SAM2^, counts 69 proteins and is represented by the RepSA protein of pSAM2. The second subfamily, RepSA^MR2^, counts 30 proteins and is represented by the RepSA protein of pMR2 (Figure 4A). Interestingly, all RepSA proteins encoded by *Streptomycineae* AICEs belong to the RepSA^SAM2^ subfamily. RepSA proteins encoded by Mmu2220/Mmul0108 and Avis01490 do not seem to belong to either subfamily; instead they cluster into a distant monophyletic clade of *Actinomycineae* replication initiator proteins.

Replication initiator proteins from distant RCR replicons (i.e., bacteriophages, plasmids, eukaryotic viruses) contain three conserved amino acids motifs [38], [39]. Motif 1 was initially of unknown function. Motif 2 contains a HUH motif (histidine-hydrophobic-histidine) important for metal-binding whereas motif 3 contains the catalytic tyrosine residue. Motif 3 was used by Koonin and Ilyina [39] to define 2 superfamilies of RCR replication initiator proteins; superfamily I contains two conserved tyrosine residues whereas superfamily II contains only one. Based on the conservation of these motifs, the pSAM2 RepSA protein was initially found to be related to replication initiator protein RepA of coliphage ϕX174 by Hagège et al. [17]. Later te Poele et al. [9] reported that the replicon pSAM2 belongs to the pC194 family based on the conserved motifs in RepSA and nick site at *dso*. To further examine the functional relatedness of the RepSA-like proteins of the AICEs detected in our analysis, we generated amino acid logo sequences for the 3 conserved motifs. While the RepSA^SAM2^ and RepSA^MR2^ protein motifs 2 and 3 exhibit some similarity with the ϕX174 Rep family, they clearly diverge at specific positions (Figure 4B). Solely based on motif 3, RepSA^SAM2^ proteins are clearly related to the ϕX174-group of RCR initiators defined by Wigel and Seitz [40] while the RepSA^MR2^ proteins would define an entirely new group of RCR initiators. This hypothesis is supported by motif 1 of the RepSA^MR2^ group which clearly differs from motif 1 of ϕX174 or pC194, while motif 1 of RepSA^SAM2^ group is very similar to these RCR initiators (Figure 4B). A sequence analysis of other RCR initiator proteins and their putative DNA binding sequences, albeit from the distant plant infecting geminiviruses family, suggests that motif 1 is involved in the *dso* binding region specificity [41], [42]. Consequently, we predict that RepSA proteins cut the *dso* nick region using a similar mechanism but the RepSA^MR2^ would recognize discrete *dso* binding regions. Such recognition differences may facilitate the coexistence of similar yet different AICE within the same genome.

**Figure 5:**
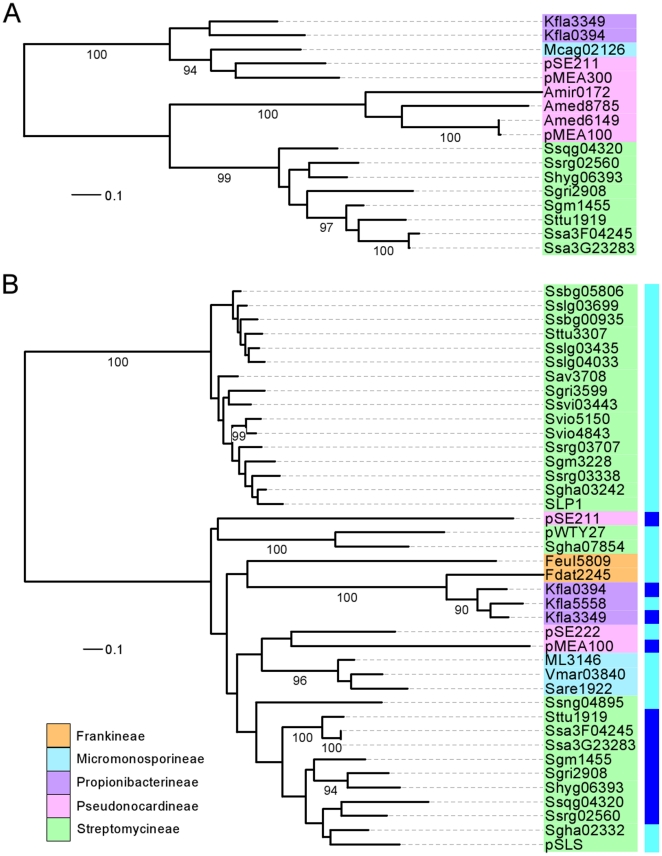
Phylogenetic trees of putative replication-associated proteins identified in putative AICEs. (A) RepAM (DUF3631) domain-containing proteins. (B) Prim-Pol (bifunctional DNA primase/polymerase) domain-containing proteins Label colors indicate the suborders of Actinomycetales within which the AICEs were found (see legend). The color strip in panel B indicates the type of associated replication protein: dark blue, RepAM; light blue, RepPP.

Other less-well characterized proteins likely involved in AICE replication were also sought for in order to unearth additional putative AICEs. The RepAM proteins, first demonstrated to be responsible for autonomous replication in AICE pMEA300 from *Amycolatopsis methanolica* [9], are predicted to function as RCR initiator proteins. Although they lack similarity to other known replication initiator protein, their putative nick sites were shown to be similar to those of the pC194 family of RCR plasmids [9]. Identification of 14 new AICEs relying on RepAM proteins reveals such AICEs are not constrained to the *Pseudonocardinae* unlike previously reported [9] (Figure 5A). Phylogenetic analysis revealed 3 clades of RepAM initiators, one of which grouping AICEs only found in *Streptomyces*. Interestingly, RepAM initiator genes of all three clades are occasionally associated with a gene coding for a Prim-Pol domain-containing protein (Figure 5B and data not shown).

Prim-Pol proteins could be involved in AICE replication as well and were identified within one of the two region important for the replication of AICE SLP1 from *S. coelicolor* A3(2) [43]. This region contains a small operon consisting of a Prim-Pol gene, SCO4617, followed by a putative replication gene, SCO4618, different from *repAM*. Our analysis revealed 40 AICEs encoding a Prim-Pol domain-protein (Figure 3A). 12 of these AICEs were found to bear a *repAM* gene immediately adjacent and 16 were found to carry a SCO4618-like gene (Figure 3B). Two ICEs, Sgha02332 and Fdat2245, were found to respectively encode a Poxvirus_D5 (PF03288, PF08706) domain- and a DNA_pol_A (PF00476) domain-containing protein. Other proteins encoded by genes associated to Prim-Pol genes in the remaining AICEs do not exhibit any similarity with any replication or DNA processing proteins known to date, nor contain domains related to this function (data not shown). Yet together with the Prim-Pol genes, these genes likely are unforeseen AICE replication modules.

AICEs replication modules are diverse. Interestingly, none of them seem to be exclusive to phylogenetically related bacteria. Consequently, some of these mobile elements may have been exchanged between distant bacteria. Additionally, AICEs from distant bacteria containing similar replication modules may have arisen from distinct recombination events with other mobile elements (e.g., plasmids, phages), although this seems less likely.

### AICE conjugation

Unlike most known conjugation systems, AICEs and *Streptomyces* conjugative plasmids transfer as double stranded circular DNA molecules and do not require a genuine T4SS [9]. AICEs conjugative transfer seems to only require a single transfer protein, Tra or TraB, which recognize and bind the *cis*-acting locus (*clt*) [44]. Recently, these Tra proteins were shown to be structurally and functionally related to septal DNA translocators of the FtsK/SpoIIIE family, gathering into hexameric channel structures and forming pores in lipid bilayers [44].

**Figure 6:**
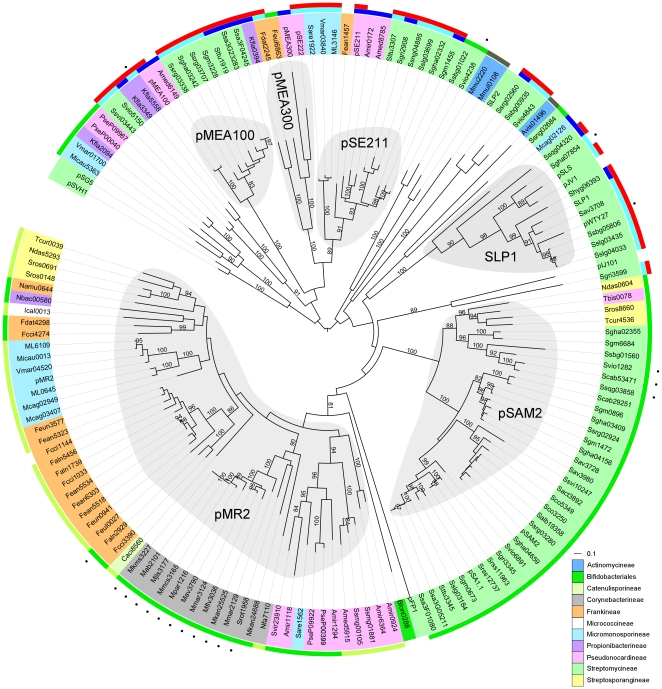
Phylogenetic tree of proteins containing an FtsK/SpoIIIE domain (Tra) found in AICEs. Sub-families were defined as clades containing at least 4 members with a distance cut-off of 2 and with a >80 bootstraps support. Label colors indicate the suborders of Actinomycetales within which the AICEs were found (see legend). Outer color strips indicate AICEs encoding Prim-Pol (red), RepAM (dark blue), RepPP (light blue), and RepSA^SAM2^ (green), RepSA^MR2^ (light green) and other RepSA-like (dark green) replication proteins. Black dots indicate AICEs encoding serine site-specific serine recombinases.

AICEs Tra proteins identified by our analysis clustered in several monophyletic clades (Figure 6). The pSAM2 and pMR2 clades, named after the eponym elements, contain the largest number of AICEs, which interestingly all encode RepSA-like RCR initiators. Three other clades, pMEA100, pSE211 and SLP1, each groups a smaller number of AICEs, which exclusively rely on RepAM and/or Prim-Pol proteins for replication. The pSAM2 and pMR2 clades are devoid of Tra protein encoded by *Streptomyces* conjugative plasmids (pSG5, pSVH1, pJV1, pIJ101, pJV1, pFP1 and SLP2), which were also included in our phylogenetic analyses. This suggests that exchanges of genetic material between AICE and *Streptomyces* conjugative plasmids are rare events. A noticeable exception is the Tra protein of pJV1, which belong to the SLP1 clade. pJV1 Tra is very similar to the Tra protein of AICE pSLS (76% identity). In addition to harboring orthologous intermycelial transfer genes, pJV1 and pSLS also carry similar intramycelial spread genes (*spdB123*) contiguous to *tra* (Figure S1). However, plasmid pJV1 does not code for an integrase and does not code for Rep proteins similar to those encoded by pSLS. This example clearly indicates that some of the AICEs and conjugative plasmids could have arisen from common ancestors and/or have exchanged functional modules.

Conjugative plasmids in *Streptomyces* have been shown to mediate chromosomal gene transfer. pIJ101-induced mobilization of chromosomal genes in *Streptomyces lividans* has been shown to occur without prior plasmid integration into the chromosome [45]. Recently, Vogelmann et al [44] reported that TraB of the *Streptomyces venezuelae* conjugative plasmid pSVH1 binds several *clt*-like regions in the whole chromosome *of S. coelicolor* suggesting a yet elusive mechanism that likely involves Tra binding onto *clt*-like sequences on the chromosome eventually promoting transfer of chromosomal DNA fragments into the recipient cells. Consequently, the numerous Tra-dependent AICEs identified herein represent a huge potential for exchange of chromosomal genes within the *Actinomycetales* order in addition to the AICE-borne cargo of auxiliary genes.

### Identification of putative T4SS-based ICEs in the genomes of actinobacteria

Furthermore, we also looked for putative ICEs relying on a T4SS-like conjugative machinery similar to the one encoded by conjugative elements of Gram-negative bacteria [14] and of Firmicutes such as ICE*Bs1* from *Bacillus subtilis* [46] or the enterococcal plasmid pIP501 [47]. For that purpose, in addition to an *int* gene, we sought for (i) a gene coding for a putative T4CP, (ii) a gene coding for a VirB4/TraC-like T4SS component, and (iii) a gene coding for a putative DNA relaxase (Mob) (Table 1). T4CP proteins are VirD4 homologs of the *Agrobacterium* T-DNA transfer system, which is a distant relative of the FtsK/SpoIIIE domain [48]. VirB4/TraC-like T4SS components usually have ATPase activity and are involved in the assembly of the pilus in Gram-negative bacteria [13]. Relaxases have been shown to be related to several types of DNA-processing proteins including RCR proteins [14], [49]. For this study, we limited our analysis to relaxases of the MOB_F_ family (TrwC relaxase domain) whose members are well represented in the Actinobacteria [49]. With a few known exceptions [50], [51], it is generally assumed that such ICEs do not replicate autonomously. Therefore the presence of a replication initiator protein was not considered as a requirement for this identification as this role can be eventually played by the Mob protein [50]. Based on these criteria, we identified 17 putative ICEs in all major Actinobacteria suborders (Table S2). Among them, one was found in *Frankia* sp. Cci3 stain. Analysis of the molecular structure of Fcci3350 revealed that this ICE encodes a lantibiotic dehydratase-like protein. Proteins from this family participate to the biosynthesis of lantibiotic, a ribosomally synthesised antimicrobial agent [52]. This T4SS-like ICE also encodes genes that may participate or limit inter-bacterial transfer of mobile genetic elements (Table S3). Several components of a putative CRISPR-Cas system, an RNA-based immune system targeting bacteriophages and plasmids in bacteria [53], are encoded by Fcci3350. However, this system is likely not functional as shown for the linear plasmid pSHK1 from *Streptomyces* [54]. Additional characterization of these new ICEs is beyond the scope of this article and will be the topic of another publication.

### AICEs and related elements in *Frankia*: general structure, synteny and gene conservation

Frankiae and their symbioses are present on all continents except Antarctica, and it is estimated that *Frankia* sp. is responsible for 15–25% of global nitrogen fixation [2], [3]. The critical role of this microorganism and its symbiosis in the global nitrogen cycle underscores the importance of deciphering their genetic inheritance, diversity, genetic plasticity and evolution. Research to date has barely touched these questions, often limited by the fastidious nature of this microorganism in the laboratory and the complete lack of genetic tools. AICEs as a whole and their building blocks, used to generate new genetic tools, could significantly facilitate the study of *Frankia* and their symbiotic interactions with plants.

**Figure 7:**
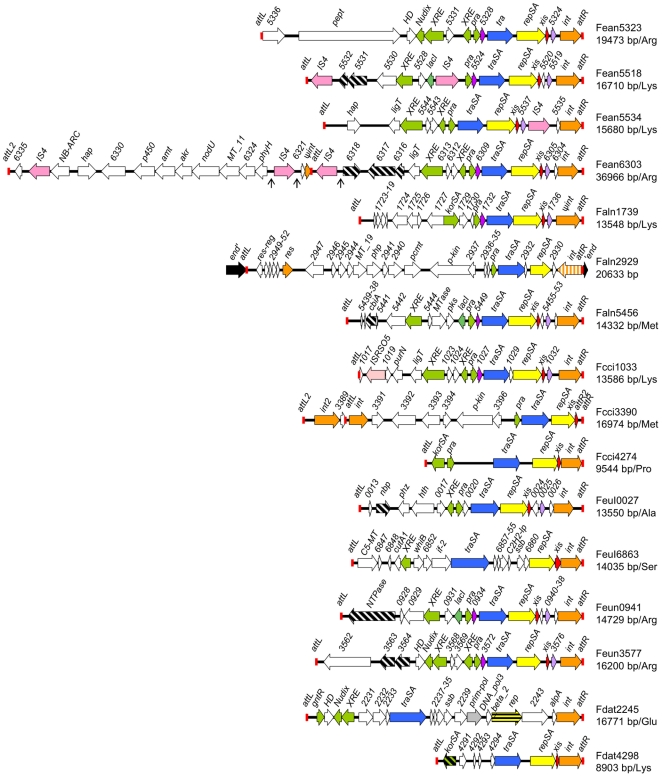
Genetic organization of putative AICEs identified in 6 *Frankia* genomes. The name, size and type of tRNA gene within which AICEs are inserted are indicated. Colour coding: orange, recombination; red, recombination directionality; yellow and gray, replication; blue, intermycelial transfer; lime green, regulation; dark green, transcriptional regulator *Lac*I; pink, IS elements; mauve and fuchsia, conserved hypothetical proteins; dashed black arrows, ORFs with G+C contents <55%. Arrows indicate the noncoding sequence regions that were deleted in order to simplify the schematic representation of Fean6303. *pept*, peptidase C14 caspase catalytic subunit P20; *HD*, metal dependent phosphohydrolase; *hap*, Peptidase S1 and S6 chymotrypsin/Hap; *LigT*, 2′–5′ RNA-ligase; *PhyH*, phytanoyl-CoA dioxygenase; *MTase*_11, methyltransferase, domain MTase_11; *NodU*, carbamoyltransferase; *PurN*, phosphoribosylglycinamide formyltransferase; *akr*, aldo/keto reductase; *amt*, aminotransferase class I and II ; *P450*, cytochrome P450; *hap*, 2-alkenal reductase; *NB-ARC*, NB-ARC domain protein; *p-kin*, putative protein kinase; *pcmt*, protein-L-isoaspartate(D-aspartate) O-methyltransferase; *php*, PHP -like protein; *MT_19*, putative S-adenosyl-L-methionine-dependent methyltransferase, *pks*, Putative modular polyketide synthase; *cbiA*, CobQ/CobB/MinD/ParA nucleotide binding domain protein; *hth*, helix-turn-helix domain protein; *phz*, phenazine biosynthesis PhzC/PhzF protein; *nbp*, nucleotide-binding protein; *ssb*, single-strand binding protein; *C2H2-lp*, zinc finger - C2H2-like protein; *if-2*, translation initiation factor IF-2; *whiB*, transcription factor WhiB; *cutA1*, divalent ion tolerance protein CutA1; *C5-MT*, C-5 cytosine-specific DNA methylase; *NTP-ase*, putative signal transduction protein with Nacht domain – NTPase; DNA_pol3_beta_2, DNA polymerase III beta subunit, central domain.

*Frankia* strains are among the species of *Actinomycetales* counting the highest number of AICEs per genome on average (Figure 2A). In this study 13 new AICEs were found in six genomes of *Frankia* in addition to the 3 AICEs identified by te Poele et al. [9] (Figure 7 and Table S1). The 16 *Frankia* AICEs have an average G+C content of 68%; yet a subset carries genes with a G+C content below 55% (dashed black arrows in Figure 7) suggesting that significant amounts of genetic material have been acquired outside the Actinobacteria. The synteny between the 16 *Frankia* AICEs was examined to better understand the relationships linking AICEs found in the same or different *Frankia* strains. This sequence analysis revealed a clear segregation of AICEs into three families (Figure 7 and S2).

The first family regroups ten AICEs (Fean5323, Fean5518, Fean5534, Fean6303, Faln1739, Faln5456, Fcci1033, FeuI0027, Feun0941 and Feun3577) that are characterized by a conserved structural module containing *pra*, *tra*, *rep*, *xis* and *int* (Table S4). Phylogenetic analysis showed that all encode a pMR2-type Tra protein and a RepSA^MR2^ RCR initiator protein (Figure 4 and 6). Although *int* was found to be the most divergent gene (Figure 3B), these observations suggest that these 10 AICEs all derive from a common ancestor. Interestingly, Xis and Pra encoded by AICEs originating from the same strain share up to 97% identity suggesting potential cross-talks between elements of the same family. For instance, transactivation of excision and conjugative transfer was reported in cells harbouring multiple copies of the ICE Tn*916* from *Enterococcus faecalis* [55]. Likewise, coordinated induction of the lytic cycle of Gifsy prophages was recently reported in polylysogenic strains of *Salmonella* [56].

The second family of AICEs includes the two smallest AICEs identified in *Frankia*, Fcci4274 and Fdat4298. While both encode a pMR2-type Tra protein, unlike AICEs of the previous family, both encode a RepSA^SAM2^ RCR initiator (Figure 4 and 6) suggesting that module exchange took place between AICEs.

The third family includes Faln2929 and Fcci3390. Like AICEs of the second family, both elements retain pMR2-type *tra* genes and RepSA^SAM2^ RCR initiator *rep* genes. However, they encode completely different *int* genes (Figure 3, 4 and 6). In Faln2929, *int* encodes a serine recombinase, which is convergent with *tra* and *rep*. Fcci3390 appears to correspond to a circular permutation of the classical AICE *att*-*tra*-*rep-xis*-*int*-*att* structure into an *att*-*int-tra-rep*-*xis*-*att* structure.

Finally, FeuI6863 and Fdat2245 do not seem to share any extended sequence identities with each other or with any AICEs of the 3 previous families. While the Tra proteins encoded by these two ICEs are distantly related to those encoded by pMEA300 and pSE222, they encode different replication initiator proteins and Fdat2245 is the only *Frankia* AICE encoding a Prim-pol replication protein (Figure 4, 5 and 6). Altogether, these observations suggest that FeuI6863 and Fdat2245 are recent acquisitions in *Frankia* species.

Fean1457, Fcci0407, Fcci1144 and Feul5809 form a group apart as they lack either a *tra* or *rep* gene (Figure S3 and Table S5). Therefore, this group may be considered as remnant AICEs or mobilizable integrating elements.

### Integration and excision of *Frankia* AICEs

With the exception of Faln2929, which encodes a serine recombinase and disrupts a gene encoding a putative endonuclease (Fraal2928, data not shown), all *Frankia* AICEs are site-specifically integrated into the 3′ end of tRNA genes, a process likely mediated by the tyrosine recombinase that they encode (Figure 3 and 7). These integrases grouped within pSE211, Fean5518, pSA1.1, Fcci3330 and SLP1 sub-families (Figure 3B). While integration of AICEs is mediated by the integrase alone, excision requires in most cases the concerted expression of a recombination directionality factor (RDF), usually called excisionase or Xis protein [57], [58]. All the AICEs identified but Faln 2929 carry a gene coding for a putative RDF (Figure 7).

Most *Frankia* AICEs exhibit the typical *att*-*tra*-*rep*-*int*-*att* structure, with a few exceptions. Fean5518 and Fean5534 are integrated in a tandem fashion into a tRNA-Lys gene in *Frankia sp*. EAN1pec. Such organization is also found in *Streptomyces avermitilis* for the AICEs Sav3708 and Sav3728 (te Poele et al. 2008 and this study), which are also integrated in tandem fashion in a tRNA-Arg gene. Interestingly, while Fean5518 and Fean5534 encode two closely related integrases, Sav3708 and Sav3728 encode integrases belonging to two different sub-families (Figure 3B). Another anomalous organization is found for Fean6303 which contains three direct repeated sequences extending beyond the 3′ end of the tRNA-Arg gene. Two of these repeated sequences correspond to two different *attL* sites, *attL* and *attL*2, and the third one is *attR* (Figure 7). The first *attL* (81 bp) defines, together with *attR*, an AICE 5 kb shorter than the structure previously described by te Poele et al. [9]. These findings suggest that Fean6303 is able to excise as two different circular molecules of 15.3 kb and 32.3 kb. A similar organization was reported for ICEs found in the lactic acid bacteria *Streptococcus thermophilus* [59]. Finally, Fcci3390 bears two different *int* genes separated by an *attL* site and a gene coding for a hypothetical protein. Interestingly, unlike most AICEs, these two *int* genes are located upstream of the *tra* gene instead of downstream of *rep*.

Excision of ICEs from the chromosome is the first step preceding their conjugative transfer. Together, the integrase and the RDF Xis catalyze a site-specific recombination event between the *attL* and *attR* attachment sites flanking the ICE to generate an *attB* site on the chromosome and an *attP* site on the circularized ICE. The latter can readily be detected using a nested PCR assay. To investigate whether the putative AICEs detected in the *Frankia* genomes were functional mobile elements, we carried out nested PCR experiments to detected *attP* for all of the putative AICEs detected in *Frankia* strains ACN14a, Ean1pec and Cci3. Nine out of ten tested AICEs were found to excise when the strains were grown in liquid cultures. Excision of Fcci4274 could not be detected in our assay.

**Figure 8:**
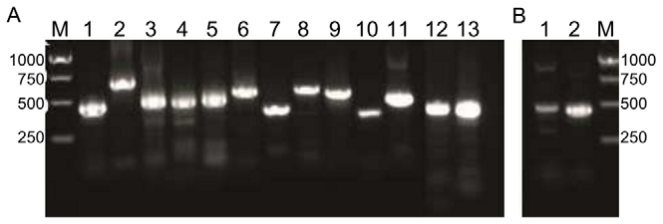
Detection of excision of putative *Frankia* AICEs *in vitro* and *in planta*. Formation of *attP* sites was detected by nested PCR in circular excised elements. (A) Detection *in vitro*. 1, Fean1457; 2, Fean5323; 3, Fean5518; 4, Fean5534; 5, Fean5518–5534; 6, Fean6303; 7, Fean6303–6336; 8, Faln1739; 9, Faln5456; 10, Fcci0407; 11, Fcci1033; 12, Faln2929; 13, Fcci3388. (B) Detection *in planta*. 1, Faln1739; 2, Faln5456. M: marker DNA fragments.

Interestingly, Fean5518 and Fean5534, which are inserted in a tandem fashion, were found to excise both independently and associatively within a large ∼31.8 kb circular molecule. Fean6303 was found to be able to excise by recombination between *attR* and either *attL* (15.3 kb form) or *attL2* (32.3 kb form) (Figure 8A, lanes 6 and 7). Similar observations have been reported for ICE*St1* and related ICEs from *S. thermophilus* [59] as well as for the High Pathogenicity Island (HPI) in ICE*Ec*1 from *Escherichia coli* [60].

Surprisingly, excision of Faln1739 was detectable despite the absence of a functional recombinase (Figure 8A, lane 8). The *int* gene of Faln1739 encodes a predicted truncated protein due to the presence of a stop codon at position 291. Another integrase encoded by the genome of ACN14a could mediate the integration and excision of this AICE; yet we failed to detect any closely related *int* gene in this genome. Furthermore, no mutation was detected in the tRNA genes for Tyr or Gln codons or other tRNA genes, ruling out the involvement of a suppressor tRNA in the translation of this gene into a functional protein. We hypothesize that slippage of the ribosome through the TAA stop codon during translation of Faln1739′s *int* allows sufficient production of a functional integrase.

Excision of Fcci3390 was observed only for the segment located between *attL* and *attR* (Figure 8A, lane 13). Phylogenetic analysis showed that the two integrases encoded by this AICE (see Fcci3388 and Fcci3390 in figure 3B) belong to two different sub-families. Excision of the segment located between *attL2* and *attR2* which should be mediated by *int2* is likely prevented by the lack of a cognate RDF.

Excision of Faln2929, which encodes a serine recombinase but no identifiable RDF, was also detected. This suggests that this recombinase is able to catalyze both integration and excision of this AICE without any requirement for an auxiliary AICE-encoded Xis protein. A similar observation has been reported for *Streptomyces* phage BT1 [61].

Finally, excision of Faln1739 and Faln5456 was also detected in *Alnus viridis* ssp. *crispa* nodules infected by *Frankia alni* ACN14a and grown in controlled laboratory conditions (Figure 8B, lanes 1 and 2). These results demonstrate the possibility of genetic exchange *in planta* involving *Frankia* and other endophytes occupying this same, narrow, ecological niche.

### Putative regulatory functions in *Frankia* ICEs

Conjugative transfer of most natural ICEs identified to date is tightly regulated and can be induced by ICE-specific environmental stimuli, such as the presence of antibiotics (tetracycline), DNA-damaging agents (UV light, mitomycin C) or quorum sensing [11]. These diverse mechanisms of induction employ a vast array of ICE-encoded regulator proteins. Some of these proteins can interact positively or negatively with other mobile genetic elements [62]. *Frankia* AICEs also include putative regulators such as orthologs of Pra encoded by pSAM2 as well as putative proteins containing XRE or GntR domains. In pSAM2 from *Streptomyces ambofaciens*, *int*, *xis* and *repSA* form an operon that is positively regulated by the product of *pra* [16]. All but three *Frankia* AICEs carry a *pra* ortholog.

GntR-family transcriptional regulators encoded by Faln1739, Fcci4274, Fdat4298, and Feul5809 are related (35%–48% identity) to KorSA, a protein encoded by pSAM2. KorSA belongs to a *kil*-*kor* system and negatively regulates the replication and transfer of pSAM2 by repressing *pra* [63]. Interestingly, the gene *korSA* of Fdat4298 has a low G+C content compared to the neighbouring ORFs suggesting that this gene had been acquired from bacteria with lower G+C content.

Eleven out of the sixteen studied AICEs contain two different types of ORFs coding for XRE-family transcriptional regulators (Figure 7). XRE proteins have an N-terminal helix-turn-helix DNA-binding motif and belong to the xenobiotic response element family of transcriptional regulators. The first type of gene, mostly located near *pra* encodes small (129 to 180 amino acid residues) XRE proteins. The second type encodes larger proteins (281 to 441 amino acid residues) sharing 27–36% identity with NsdA proteins from various *Streptomyces* strains, such as SCO5582 from *S. coelicolor*. SCO5582 is a negative regulator of antibiotic production and sporulation [64]. FeuI6863 encodes a putative WhiB-like transcription factor. WhiB has an essential role in sporulation in *S. coelicolor* [65].

The presence of such transcriptional regulators in AICEs suggests that acquisition of specific AICEs by *Frankia* strains could eventually alter particular pathways or functions in *Frankia* such as secondary metabolite production, establishment of symbiosis or nitrogen fixation.

### Auxiliary functions encoded by AICEs in *Frankia*

Examination of the general molecular structure of AICEs from the six strains of *Frankia* revealed the presence of an impressive number of genes coding for hypothetical proteins. A search with batch Blast combined with an HMMsearch against all Pfam-A families from Pfam 25.0 database allowed us to attribute putative functions to a significant number of hypothetical genes (Table S6). Two main groups of genes carried by *Frankia* AICEs can be distinguished based on the predicted functions: (i) genes coding for proteins involved in nucleic acids and amino acids metabolism and (ii) genes coding for adaptive functions.

In the first group, a cluster of 3 genes is shared by four *Frankia* AICEs and by Sco5349, pMEA100 and pSE211. This gene cluster contains *mdp* (metal-dependent phosphohydrolase), *nud* (Nudix hydrolase) and *xre* (XRE-family transcriptional regulator). Metal-dependent phosphohydrolases are HD hydrolases usually involved in the nucleic acid metabolism and signal transduction in bacteria and archaea [66]. Nudix hydrolases such as MutT in *E. coli* have the ability to degrade potentially mutagenic oxidised nucleotides [67], [68]. The Nudix hydrolase Pif of pSAM2 is involved in conjugal immunity [27]. Fcci1033 encodes a predicted phosphoribosylglycinamide formyltransferase (EC:2.1.2.2). Proteins from this family are involved in the biosynthesis of secondary metabolites and nucleotide metabolism.

The second group of genes code for adaptive functions such as heavy metals resistance, antibiotic biosynthesis and response to different environmental stressors. Fean6303 encodes a carbamoyltransferase belonging to the same family as the *Rhizobium* protein NodU, which is involved in the synthesis of nodulation factors, and CmcH, a protein from *Nocardia lactamdurans*, which is involved in the biosynthesis of the antibiotic cephamycin (Coque et al. 1995; Jabbouri et al. 1995).

Faln5456 encodes a 150-amino acid protein with predicted modular polyketide synthase activity. In streptomycetes, polyketide synthases participates to the synthesis of molecules with antibiotic and antifungal properties [69], [70]. Interestingly, Faln5456 and Faln5323 encode two distantly related putative S-adenosyl-L-methionine-dependent methyltransferases. Such enzymes are known to be important for polyketide activation [69]. Finally, FeuI6863 encodes a putative CutA1-like divalent ion tolerance protein, which may be involved in resistance toward heavy metals.

### Concluding remarks

Together with other mobile elements such as plasmids and prophages, ICEs are important catalysts for bacterial genomes evolution. In addition to their own dissemination, ICEs of different families are also mobilizing chromosomal DNA by an increasing number of ways such as *hfr*-like transfer [71], mobilization of related genomic islands [62], [72] and possibly by recognition of *clt*-like regions by Tra proteins in *Streptomyces* [44], [45]. In contrast to T4SS ICEs which were recently determined to be the most abundant conjugative elements in prokaryotes [73], AICEs have received little attention. The systematic *in silico* identification of 144 AICEs in the whole Actinobacteria phylum reveals the diversity of possible canonical AICEs modules (integration, replication, transfer) and but mostly their surprising combination plasticity. As an example, Int-Tyr family proteins cluster in several sub-families which are not necessarily associated to the same replication protein families. Both tyrosine and serine integrase family proteins promote the specific integration in a vast array of different sites, mostly tRNA genes. These elements are promising substrates for the development of molecular genetic tools for gene expression as well as gene interruption. Moreover, the observation of many AICEs in the same genome, as well as putative T4SS based ICEs in Actinobacteria, further expands the possibility for large genome modification by these possible molecular tools in diverse species, many of which, such as *Frankia* spp, are currently lacking effective genetic tools for their study.

A total of sixteen different AICEs were identified in the genomes of six *Frankia* strains. Nine of these AICEs were shown to excise *in vitro* from the chromosome by site-specific recombination, the initial prerequisite step for their conjugation. Additionally, excision *in planta* of two of these AICEs was shown to occur while in symbiotic association in root nodules of *Alnus viridis*. *Frankia* AICEs harbor diverse sets of genes that do not seem to be involved in their mobility, but rather encode for putative proteins likely involved in AICEs compatibility and exclusion, nodulation or secondary metabolism. Although the possible advantages conferred by AICE-borne genes have yet to be determined, our findings suggest that AICEs play an adaptive role and establishes new grounds for evolutionary and molecular study of Actinobacteria.

## Materials and methods

### Bioinformatic analyses

The predicted proteomes of 275 genomes (Table S7) and 176 (Table S8) plasmids of Actinobacteria available in June 2011 were recovered from the RefSeq database [21] and analyzed to identify putative ICEs. Integrases, and putative transfer and replication proteins were sought for in these proteomes using profile hidden Markov models (HMMs) with HMMsearch from the HMMER v3.0 software package [74]. The HMM profiles that were used for data mining were recovered from the Pfam 25.0 database [75] and are summarized in Table 1. Since no HMM profile for the RepSA-like replication proteins was available at the time of this study, it was built using HMMbuild from the HMMER v3.0 software package from a C- and N-terminal trimmed multiple sequence alignment of the RepSA proteins described by te Poele *et al* [9] using MUSCLE multiple sequence alignment software [76] (File S1). Maximum-likehood phylogenies of AICE conserved proteins were generated using the PhyML v3.0 program [77] with the LG substitution model. Tree topologies were optimized by PhyML using both the NNI and SPR methods and the starting tree was estimated using BioNJ. Branch support of the phylogenies was estimated using non-parametric bootstrap (100 replicates). Phylogenetic analyses were computed from reliable amino acid alignments built by MUSCLE. Removal of poorly aligned regions from amino acid alignments was carried out by trimAl v1.2 software using the automated heuristic approach [78] prior to phylogenetic analyses. Phylogenetic trees were viewed using iTOL v2 [79] and are available as a shared project on the iTOL website (http://itol.embl.de/shared/VincentBurrus). The search for tRNA genes, located near integrase genes, was performed using tRNAscan-SE webserver [80].

Large genome segments (30 to 60 kbp) containing at least a site-specific recombination gene and a DNA translocation and replication gene were analysed with Yass [81] to identify the direct repeats of the *attL* and *attR* attachment sites flanking *Frankia* ICEs. The direct repeat sequence corresponding to the *attR* site was usually found to be the 3′ end of a tRNA-encoding gene. Predictions of domains and functions of *Frankia* ICE-encoded proteins were determined using HMMsearch against all Pfam-A families from Pfam 25.0 database. Comparative analyses of AICEs were carried out using Mauve [82].

### Bacterial strain and culture methods

*Frankia alni* sp. ACN14a was isolated in 1982 from nodules of *Alnus viridis* subsp. *crispa* plants in Tadoussac, QC, Canada [83] (catalog registry number ULQ010201401) [84]. *Frankia* sp. EAN1pec was isolated in 1978 from nodules of *Elaeagnus augustifolia* growing in Ohio, U.S.A. [85] (catalog registry number ULQ13100144). *Frankia* sp. CcI3 was isolated in 1983 from nodules of *Casuarina cunninghamiana* (MA, U.S.A.) (catalog registry number HFP020203) [86]. *Frankia alni* ACN14a and *Frankia* sp. Cci3 were grown at 30°C (obscurity, static) in BAPS medium: propionate-BAP medium [87] supplemented with 5 g/L sodium succinate. *Frankia* sp. EAN1pec was grown at 25°C (obscurity, static) in MP medium supplemented with 20 mM fructose as described by Tisa and Ensign [88].

### DNA extraction from liquid culture and nodules

Total genomic DNA from *Frankia* strains grown in liquid cultures or present in *Alnus viridis* subsp. *crispa* nodules grown in controlled laboratory conditions was purified using REDExtract-N-Amp™ Plant or Seeds PCR Kit (Sigma-Aldrich; St-Louis, MO, USA).

### ICE excision assays

ICE excision was determined by nested PCR [62]. Primers used for the detection of circularized forms of ICEs are described in Table S9. REDExtract-N-Amp™ PCR ReadyMix™ (Sigma-Aldrich) was used to perform amplification in 20 µl reactions in the following conditions: 95°C for 5 min, 35 cycles of 95°C for 30 s, 57–67°C for 1 min (depending on the set of primers used) and 72°C for 30 s, with a final elongation step at 72° for 10 min. Then 1 µl of amplification product, obtained following the first round of PCR, was used for a second round of PCR using different sets of primers identified as inner primers in Table S9. PCR conditions were the same as described above. Amplicons from the second round of PCR were sequenced by Genome Quebec Innovation Center of McGill University (Montreal, QC, Canada).

## Supporting Information

Figure S1 **Selected AICEs and plasmids synteny.** The synteny maps were generated by Mauve. Colored blocks correspond depict collinear and homologous sequences or regions between AICEs DNA sequences. A similarity profile is represented inside each block. The height of the similarity profile corresponds to the average level of conservation in that region of AICEs sequence. Regions outside blocks lack detectable homology among the studied AICEs.
(TIFF)

Figure S2 **Synteny of** ***Frankia*** **AICEs.** Synteny between *Frankia* AICEs was determined as indicated in Figure S2. *attL* and *attR* sites are indicated by flanking red vertical lines.
(TIFF)

Figure S3 **Genetic organization of putative** ***Frankia*** **AICE remnants.** The genetic organisation of the elements is depicted as in Figure 7. *GT2*, putative glycosyl transferase family 2; *HATP-ase*, putative signal transduction histidine kinase; *GP49*, GP49-like protein; *apc5*, anaphase-promoting complex subunit 5; *s-kin*, serine/threonine protein kinase; *lt*, lytic transglycosylase; *PALP*, pyridoxal-5′-phosphate-dependent protein subunit beta.
(TIFF)

File S1 **RepSA HMM.**
(HMM)

Table S1 **AICEs identified in this study.**
(XLS)

Table S2 **T4SS ICEs identified in this study.**
(XLS)

Table S3 **Frankia T4SS ICE gene content.**
(DOC)

Table S4 **Frankia AICEs orthologs.**
(XLS)

Table S5 **Predicted functions of putative proteins encoded by** ***Frankia*** **remnant AICEs or mobilizable integrating elements.**
(DOC)

Table S6 **Predicted functions of putative proteins encoded by** ***Frankia*** **AICEs.**
(DOC)

Table S7 **Actinobacterial genomes analysed in this study.**
(XLS)

Table S8 **Actinobacterial plasmids analysed in this study.**
(XLS)

Table S9 **Primers used in this study.**
(DOC)
