# Supplementary figures and images for: Uncovering the Prevalence and Diversity of Integrating Conjugative Elements in Actinobacteria
Source: PLoS One. 2011 Nov 16;6(11):e27846. doi: 10.1371/journal.pone.0027846 (PMC3218068; doi:10.1371/journal.pone.0027846)

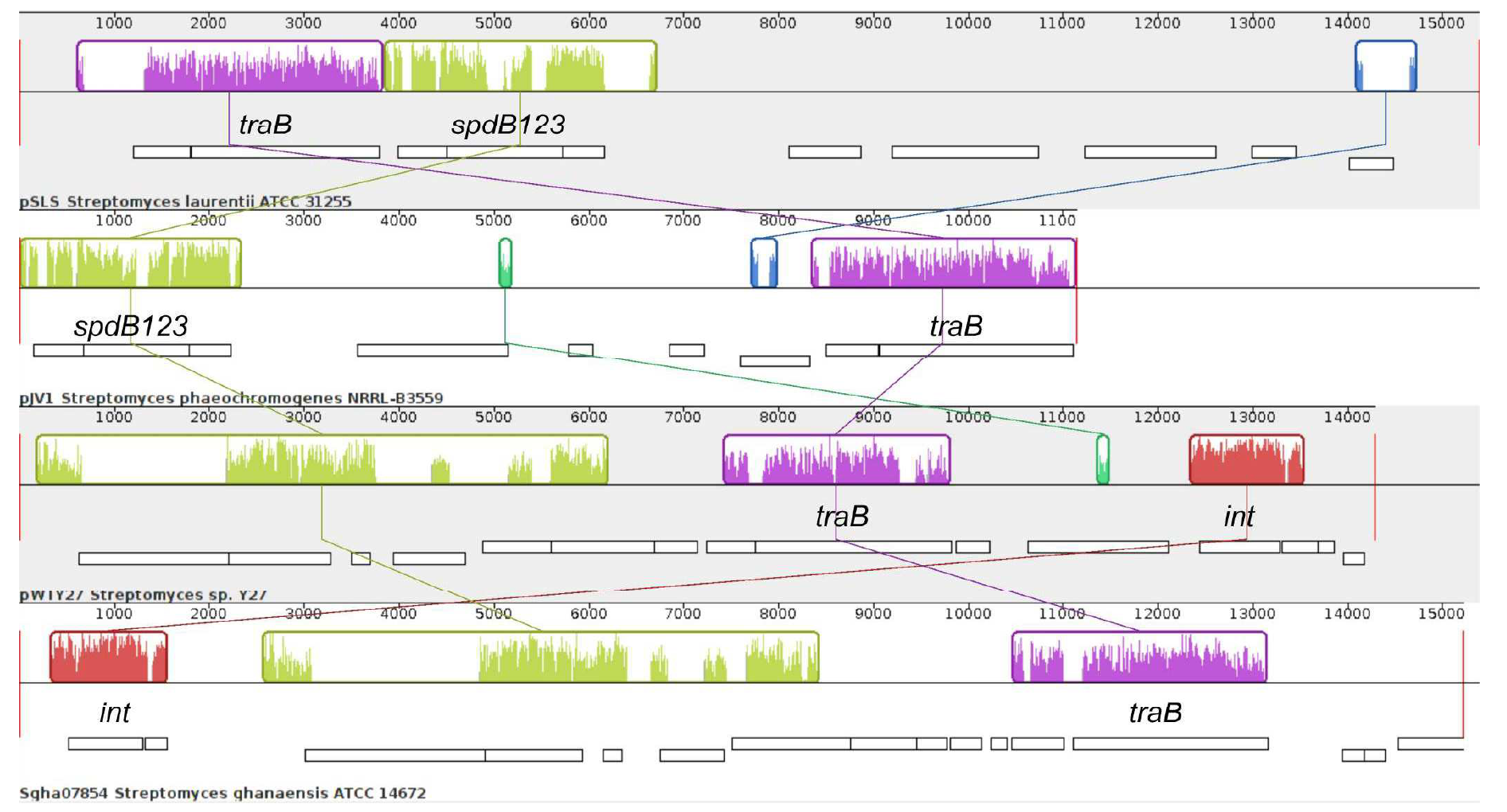

Supplement: Figure S1 — Selected AICEs and plasmids synteny. The synteny maps were generated by Mauve. Colored blocks correspond depict collinear and homologous sequences or regions between AICEs DNA sequences. A similarity profile is represented inside each block. The height of the similarity profile corresponds to the average level of conservation in that region of AICEs sequence. Regions outside blocks lack detectable homology among the studied AICEs. (TIFF) [file pone.0027846.s001.tiff]

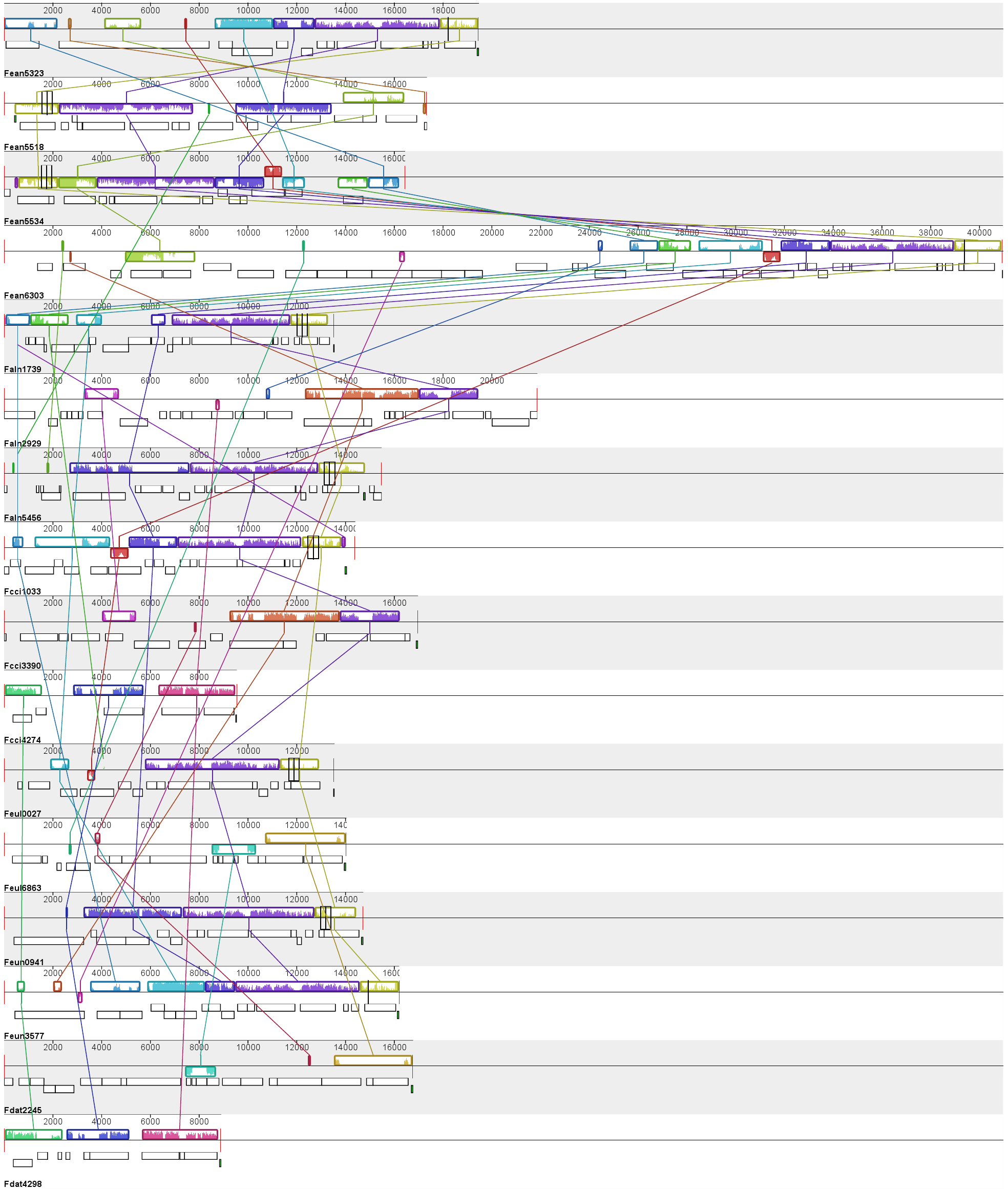

Supplement: Figure S2 — Synteny of Frankia AICEs. Synteny between Frankia AICEs was determined as indicated in Figure S2. attL and attR sites are indicated by flanking red vertical lines. (TIFF) [file pone.0027846.s002.tiff]

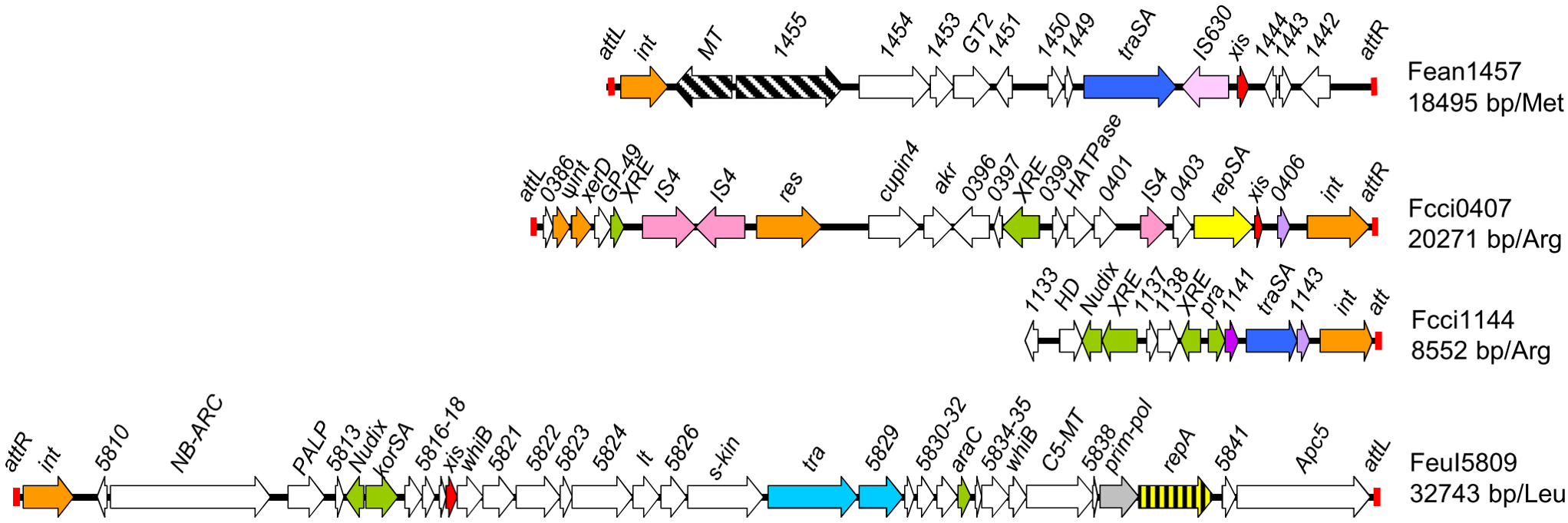

Supplement: Figure S3 — Genetic organization of putative Frankia AICE remnants. The genetic organisation of the elements is depicted as in Figure 7. GT2, putative glycosyl transferase family 2; HATP-ase, putative signal transduction histidine kinase; GP49, GP49-like protein; apc5, anaphase-promoting complex subunit 5; s-kin, serine/threonine protein kinase; lt, lytic transglycosylase; PALP, pyridoxal-5′-phosphate-dependent protein subunit beta. (TIFF) [file pone.0027846.s003.tiff]
