# Supplementary material for: Uncovering the Prevalence and Diversity of Integrating Conjugative Elements in Actinobacteria
Source: PLoS One. 2011 Nov 16;6(11):e27846. doi: 10.1371/journal.pone.0027846 (PMC3218068; doi:10.1371/journal.pone.0027846)
Supplement: Table S3 — Frankia T4SS ICE gene content. (DOC) [file pone.0027846.s007.doc]

Table S3. Predicted functions of putative proteins encoded by *Frankia* T4SS-like ICE.

| **AICE** | **Protein name** | **Size (aa)** | **Predicted function** |
| --- | --- | --- | --- |
| **Fcci3350** | Francci3_3297 | 162 | Hypothetical protein |
|  | Francci3_3298 | 48 | Hypothetical protein |
|  | Francci3_3299 | 569 | Hypothetical protein |
|  | Francci3_3300 | 214 | Hypothetical protein |
|  | Francci3_3301 | 602 | Hypothetical protein |
|  | Francci3_3302 | 294 | Hypothetical protein |
|  | Francci3_3303 | 517 | Hypothetical protein |
|  | Francci3_3304 | 318 | Hypothetical protein |
|  | Francci3_3305 | 690 | Type IV secretory pathway VirD4 component |
|  | Francci3_3306 | 296 | Hypothetical protein |
|  | Francci3_3307 | 389 | CHC2-type zinc finger-containing protein |
|  | Francci3_3308 | 500 | XRE family transcriptional regulator |
|  | Francci3_3309 | 121 | Hypothetical protein |
|  | Francci3_3310 | 124 | Hypothetical protein |
|  | Francci3_3311 | 363 | Hypothetical protein |
|  | Francci3_3312 | 750 | Hypothetical protein |
|  | Francci3_3313 | 433 | Transposase, IS4 |
|  | Francci3_3314 | 381 | Hypothetical protein |
|  | Francci3_3315 | 1027 | Lantibiotic dehydratase-like protein |
|  | Francci3_3316 | 431 | Protein-L-isoaspartate(D-aspartate) O-MT |
|  | Francci3_3317 | 458 | Hypothetical protein |
|  | Francci3_3318 | 258 | Glycosyl transferase family protein |
|  | Francci3_3319 | 224 | Acetyl/acyl transferase-like protein |
|  | Francci3_3320 | 261 | Putative hydrolase |
|  | Francci3_3321 | 71 | Hypothetical protein |
|  | Francci3_3322 | 480 | Putative ATP-binding protein |
|  | Francci3_3323 | 566 | Hypothetical protein |
|  | Francci3_3324 | 511 | Hypothetical protein |
|  | Francci3_3325 | 67 | Hypothetical protein |
|  | Francci3_3326 | 135 | Hypothetical protein |
|  | Francci3_3327 | 1123 | Peptidoglycan-binding LysM |
|  | Francci3_3328 | 414 | Hypothetical protein |
|  | Francci3_3329 | 367 | Hypothetical protein |
|  | Francci3_3330 | 294 | Type II secretion system protein |
|  | Francci3_3331 | 301 | Hypothetical protein |
|  | Francci3_3332 | 449 | Type II secretion system protein E |
|  | Francci3_3333 | 346 | Hypothetical protein |
|  | Francci3_3334 | 296 | Hypothetical protein |
|  | Francci3_3335 | 227 | Hypothetical protein |
|  | Francci3_3336 | 300 | Hypothetical protein |
|  | Francci3_3337 | 382 | Hypothetical protein |
|  | Francci3_3338 | 1520 | Hypothetical protein |
|  | Francci3_3339 | 245 | Hypothetical protein |
|  | Francci3_3340 | 80 | Hypothetical protein |
|  | Francci3_3341 | 751 | CRISPR-associated helicase Cas3 family protein |
|  | Francci3_3342 | 221 | Hypothetical protein |
|  | Francci3_3343 | 627 | Hypothetical protein |
|  | Francci3_3344 | 295 | CRISPR-associated Csh2 family protein |
|  | Francci3_3345 | 221 | CRISPR-associated Cas4 family protein |
|  | Francci3_3346 | 344 | CRISPR-associated Cas1 family protein |
|  | Francci3_3347 | 96 | Hypothetical protein |
|  | Francci3_3348 | 89 | Hypothetical protein |
|  | Francci3_3349 | 453 | Hypothetical protein |
|  | Francci3_3350 | 508 | Phage integrase |
