# Supplementary material for: Uncovering the Prevalence and Diversity of Integrating Conjugative Elements in Actinobacteria
Source: PLoS One. 2011 Nov 16;6(11):e27846. doi: 10.1371/journal.pone.0027846 (PMC3218068; doi:10.1371/journal.pone.0027846)
Supplement: Table S5 — Predicted functions of putative proteins encoded by Frankia remnant AICEs or mobilizable integrating elements. (DOC) [file pone.0027846.s009.doc]

Table S5. Predicted functions of putative proteins encoded by *Frankia* remnant AICEs or mobilizable integrating elements.

| **Name** | **Protein name** | **Size (aa)** | **Predicted function** |
| --- | --- | --- | --- |
| **Fean1457** | Franean1_1457 | 391 | Integrase Int |
|  | Franean1_1456 | 369 | Putative RNA/DNA methylase (MTase) |
|  | Franean1_1455 | 860 | Hypothetical protein |
|  | Franean1_1454 | 572 | Hypothetical protein |
|  | Franean1_1453 | 188 | Hypothetical protein |
|  | Franean1_1452 | 299 | Putative glycosyl transferase family 2, GT2 |
|  | Franean1_1451 | 134 | Hypothetical protein |
|  | Franean1_1450 | 120 | Hypothetical protein |
|  | Franean1_1449 | 63 | Hypothetical protein |
|  | Franean1_1448 | 741 | Transfer protein TraSA cell division FtsK/SpoIIIE |
|  | Franean1_1446 | 377 | IS630-transposase |
|  | Franean1_1445 | 89 | Excisionase Xis |
|  | Franean1_1444 | 95 | Hypothetical protein |
|  | Franean1_1443 | 98 | Hypothetical protein |
|  | Franean1_1442 | 235 | Hypothetical protein |
| **Fcci0407** | Francci3_0407 | 503 | Phage integrase, Int |
|  | Francci3_0406 | 99 | Hypothetical protein |
|  | Francci3_0405 | 63 | Excisionase, Xis |
|  | Francci3_0404 | 476 | Replication initiator protein RepSA |
|  | Francci3_0403 | 148 | Putative S-adenosyl-L-methionine-dependent methyltransferase, MTase_19 |
|  | Francci3_0402 | 216 | Transposase IS4 |
|  | Francci3_0401 | 181 | Hypothetical protein |
|  | Francci3_0400 | 203 | Putative signal transduction histidine kinase, HATPase |
|  | Francci3_0399 | 101 | Hypothetical protein |
|  | Francci3_0398 | 296 | Putative DNA-binding protein XRE family (toxin- antitoxin system - antitoxin component) |
|  | Francci3_0397 | 69 | Hypothetical protein (DUF397 domain) |
|  | Francci3_0396 | 296 | Hypothetical protein |
|  | Francci3_0395 | 231 | Aldo/keto reductase, Akr |
|  | Francci3_0394 | 406 | Cupin_4 |
|  | Francci3_0393 | 522 | Serine recombinase, Res |
|  | Francci3_0392 | 390 | Transposase IS4 |
|  | Francci3_0391 | 428 | Transposase IS4 |
|  | Francci3_0390 | 104 | Transcriptional regulator XRE family |
|  | Francci3_0389 | 130 | GP49-like protein (Plasmid toxin-antitoxin system) |
|  | Francci3_0388 | 161 | XerD recombinase |
|  | Francci3_0387 | 136 | Pseudointegrase |
|  | Francci3_0386 | 79 | Hypothetical protein (DUF433 domain) |
| **Fcci1144** | Francci3_1144 | 422 | Integrase, Int |
|  | Francci3_1143 | 99 | Hypothetical protein |
|  | Francci3_1142 | 409 | Transfer protein TraSA cell division FtsK/SpoIIIE |
|  | Francci3_1141 | 108 | Hypothetical protein |
|  | Francci3_1140 | 134 | Plasmid replication, integration and excision activator Pra |
|  | Francci3_1139 | 162 | Transcriptional regulator XRE family |
|  | Francci3_1138 | 165 | Hypothetical protein |
|  | Francci3_1137 | 91 | Hypothetical protein |
|  | Francci3_1136 | 126 | Transcriptional regulator XRE family |
|  | Francci3_1135 | 56 | MutT (NUDIX domain) |
|  | Francci3_1134 | 182 | Metal dependent phosphohydrolase (HD domain) |
|  | Francci3_1133 | 105 | Hypothetical protein |
| **FeuI5809** | FraEuI1c_5809 | 404 | Integrase family protein, Int |
|  | FraEuI1c_5810 | 82 | Hypothetical protein |
|  | FraEuI1c_5811 | 1283 | NB-ARC domain protein |
|  | FraEuI1c_5812 | 296 | Pyridoxal-5'-phosphate-dependent protein subunit beta, PALP |
|  | FraEuI1c_5813 | 66 | Hypothetical protein |
|  | FraEuI1c_5814 | 144 | MutT (NUDIX domain) |
|  | FraEuI1c_5815 | 257 | Transcriptional regulator, GntR family, KorSA1 |
|  | FraEuI1c_5816 | 140 | Hypothetical protein |
|  | FraEuI1c_5817 | 100 | Hypothetical protein |
|  | FraEuI1c_5818 | 57 | Hypothetical protein |
|  | FraEuI1c_5819 | 87 | DNA binding domain protein, excisionase family, Xis |
|  | FraEuI1c_5820 | 208 | Transcription factor WhiB |
|  | FraEuI1c_5821 | 255 | Hypothetical protein |
|  | FraEuI1c_5822 | 355 | Hypothetical protein |
|  | FraEuI1c_5823 | 97 | Hypothetical protein |
|  | FraEuI1c_5824 | 486 | Hypothetical protein |
|  | FraEuI1c_5825 | 214 | Lytic transglycosylase, LT |
|  | FraEuI1c_5826 | 206 | Hypothetical protein |
|  | FraEuI1c_5827 | 609 | Serine/threonine protein kinase, S-kin |
|  | FraEuI1c_5828 | 719 | Transfer protein Tra, FtsK/SpoIIIE |
|  | FraEuI1c_5829 | 355 | Transfer protein Tra, FtsK/SpoIIIE |
|  | FraEuI1c_5830 | 78 | Hypothetical protein |
|  | FraEuI1c_5831 | 140 | Hypothetical protein (DUF3307 domain) |
|  | FraEuI1c_5832 | 169 | Hypothetical protein |
|  | FraEuI1c_5833 | 106 | AraC family transcriptional regulator |
|  | FraEuI1c_5834 | 56 | Hypothetical protein |
|  | FraEuI1c_5835 | 218 | Hypothetical protein |
|  | FraEuI1c_5836 | 141 | Transcription factor WhiB |
|  | FraEuI1c_5837 | 545 | C-5 cytosine-specific DNA methylase, C5-MTase |
|  | FraEuI1c_5838 | 42 | Hypothetical protein |
|  | FraEuI1c_5839 | 311 | Bifunctional DNA primase/polymerase, Prim-pol |
|  | FraEuI1c_5840 | 612 | Replication initiator protein, RepA |
|  | FraEuI1c_5841 | 108 | Hypothetical protein |
|  | FraEuI1c_5842 | 1069 | Anaphase-promoting complex subunit 5, Apc5  TPR domain protein |
