# Supplementary material for: Uncovering the Prevalence and Diversity of Integrating Conjugative Elements in Actinobacteria
Source: PLoS One. 2011 Nov 16;6(11):e27846. doi: 10.1371/journal.pone.0027846 (PMC3218068; doi:10.1371/journal.pone.0027846)
Supplement: Table S6 — Predicted functions of putative proteins encoded by Frankia AICEs. (DOC) [file pone.0027846.s010.doc]

Table S6. Predicted functions of putative proteins encoded by *Frankia* AICEs.

| **AICE** | **Protein name** | **Size (aa)** | **Predicted function** |
| --- | --- | --- | --- |
| **Fean5323** | Franean1_5323 | 492 | Integrase, Int |
|  | Franean1_5324 | 99 | Hypothetical protein |
|  | Franean1_5325 | 61 | Excisionase Xis |
|  | Franean1_5326 | 572 | Replication initiator protein RepSA |
|  | Franean1_5327 | 523 | Transfer protein TraSA cell division FtsK/SpoIIIE |
|  | Franean1_5328 | 89 | Hypothetical protein |
|  | Franean1_5329 | 135 | Plasmid replication, integration and excision activator Pra |
|  | Franean1_5330 | 152 | XRE transcriptional regulator |
|  | Franean1_5331 | 153 | Hypothetical protein |
|  | Franean1_5332 | 392 | Transcriptional regulator XRE family |
|  | Franean1_5333 | 156 | MutT (NUDIX domain) |
|  | Franean1_5334 | 182 | Metal dependent phosphohydrolase (HD domain) |
|  | Franean1_5335 | 2050 | Peptidase C14 caspase |
|  | Franean1_5336 | 450 | Hypothetical protein |
| **Fean5518** | Franean1_5518 | 477 | Integrase, Int |
|  | Franean1_5519 | 99 | Hypothetical protein |
|  | Franean1_5520 | 67 | Hypothetical protein |
|  | Franean1_5521 | 63 | Excisionase, Xis |
|  | Franean1_5522 | 569 | Replication initiator protein, RepSA |
|  | Franean1_5523 | 523 | Transfer protein TraSA cell division FtsK/SpoIIIE |
|  | Franean1_5524 | 96 | Hypothetical protein |
|  | Franean1_5525 | 136 | Plasmid replication, integration and excision activator, Pra |
|  | Franean1_5526 | 462 | Transposase IS4 family |
|  | Franean1_5527 | 131 | Transcriptional regulator LacI family |
|  | Franean1_5528 | 142 | Hypothetical protein |
|  | Franean1_5529 | 327 | Transcriptional regulator XRE family |
|  | Franean1_5530 | 398 | Hypothetical protein |
|  | Franean1_5531 | 390 | Hypothetical protein (DUF262 domain) |
|  | Franean1_5532 | 184 | Hypothetical protein |
|  | Franean1_5533 | 420 | Transposase IS4 family |
| **Fean5534** | Franean1_5534 | 426 | Integrase, Int |
|  | Franean1_5535 | 69 | Hypothetical protein |
|  | Franean1_5536 | 425 | Transposase IS4 family |
|  | Franean1_5537 | 99 | Hypothetical protein |
|  | Franean1_5538 | 64 | Excisionase, Xis |
|  | Franean1_5539 | 573 | Replication initiator protein, RepSA |
|  | Franean1_5540 | 517 | Transfer protein TraSA cell division FtsK/SpoIIIE |
|  | Franean1_5541 | 134 | Plasmid replication, integration and excision activator Pra |
|  | Franean1_5542 | 126 | Transcriptional regulator XRE family |
|  | Franean1_5543 | 151 | Hypothetical protein |
|  | Franean1_5544 | 94 | Hypothetical protein |
|  | Franean1_5545 | 443 | Transcriptional regulator XRE family |
|  | Franean1_5546 | 226 | 2’-5’ RNA-ligase, LigT |
|  | Franean1_5547 | 266 | Peptidase S1 and S6 chymotrypsin/Hap |
| **Fean6303** | Franean1_6303 | 416 | Integrase, Int |
|  | Franean1_6304 | 69 | Hypothetical protein |
|  | Franean1_6305 | 99 | Hypothetical protein |
|  | Franean1_6306 | 64 | Excisionase, Xis |
|  | Franean1_6307 | 572 | Replication initiator protein, RepSA |
|  | Franean1_6308 | 519 | Transfer protein TraSA cell division FtsK/SpoIIIE |
|  | Franean1_6309 | 95 | Hypothetical protein |
|  | Franean1_6310 | 134 | Plasmid replication, integration and excision activator, Pra |
|  | Franean1_6311 | 126 | Transcriptional regulator XRE family |
|  | Franean1_6312 | 128 | Hypothetical protein |
|  | Franean1_6313 | 94 | Hypothetical protein |
|  | Franean1_6314 | 443 | Transcriptional regulator XRE family |
|  | Franean1_6315 | 226 | LigT 2'-5' RNA ligase |
|  | Franean1_6316 | 182 | Hypothetical protein |
|  | Franean1_6317 | 552 | Hypothetical protein |
|  | Franean1_6318 | 365 | Hypothetical protein |
|  | Franean1_6319 | 420 | Transposase IS4 family |
|  | Franean1_6320 | 120 | Hypothetical protein |
|  | Franean1_6321 | 79 | Hypothetical protein (DUF433 domain) |
|  | Franean1_6322 | 420 | Transposase IS4 family |
|  | Franean1_6323 | 239 | Phytanoyl-CoA dioxygenase, PhyH |
|  | Franean1_6324 | 327 | Hypothetical protein |
|  | Franean1_6325 | 403 | Methyltransferase, domain MTase_11 |
|  | Franean1_6326 | 551 | Carbamoyltransferase, CmcH_NodU |
|  | Franean1_6327 | 340 | Aldo/keto reductase, Akr |
|  | Franean1_6328 | 397 | Aminotransferase class I and II, Amt |
|  | Franean1_6329 | 423 | Cytochrome P450 |
|  | Franean1_6330 | 488 | Hypothetical protein |
|  | Franean1_6331 | 372 | 2-alkenal reductase, Hap |
|  | Franean1_6333 | 373 | NB-ARC domain protein |
|  | Franean1_6334 | 425 | Transposase IS4 family |
|  | Franean1_6335 | 144 | Tetratricopeptide repeat domain (TPR) protein |
| **Faln1739** | FRAAL 1739 | 459 | Pseudo Integrase, Int |
|  | FRAAL 1736 | 95 | Hypothetical protein |
|  | FRAAL 1735 | 61 | Excisionase, Xis |
|  | FRAAL 1734 | 567 | Replication initiator protein, RepSA |
|  | FRAAL 1733 | 535 | Transfer protein TraSA cell division FtsK/SpoIIIE |
|  | FRAAL 1732 | 104 | Hypothetical protein |
|  | FRAAL 1731 | 128 | Plasmid replication, integration and excision activator Pra |
|  | FRAAL 1730 | 69 | Hypothetical protein |
|  | FRAAL 1729 | 174 | Hypothetical protein |
|  | FRAAL 1728 | 302 | Transcriptional regulator, GntR family, KorSA |
|  | FRAAL 1727 | 349 | Hypothetical protein with DUF1152 domain |
|  | FRAAL 1726 | 88 | Hypothetical protein |
|  | FRAAL 1725 | 218 | Hypothetical protein with TauD domain |
|  | FRAAL 1724 | 305 | Hypothetical protein |
|  | FRAAL 1723 | 31 | Hypothetical protein |
|  | FRAAL 1722 | 111 | Hypothetical protein |
|  | FRAAL 1720 | 185 | Hypothetical protein |
|  | FRAAL 1719 | 36 | Hypothetical protein |
| **Faln2929** | FRAAL2929 | 502 | Serine recombinase Integrase, Int |
|  | FRAAL2930 | 72 | Hypothetical protein |
|  | FRAAL2931 | 418 | Replication initiator protein, Rep |
|  | FRAAL2932 | 58 | Hypothetical protein |
|  | FRAAL2933 | 558 | Transfer protein TraSA cell division FtsK/SpoIIIE |
|  | FRAAL2934 | 103 | Putative regulatory protein Pra |
|  | FRAAL2935 | 59 | Hypothetical protein |
|  | FRAAL2936 | 64 | Hypothetical protein |
|  | FRAAL2937 | 147 | Hypothetical protein |
|  | FRAAL2938 | 813 | Putative protein kinase, P-kin |
|  | FRAAL2939 | 340 | Protein-L-isoaspartate(D-aspartate) O-methyltransferase (PCMT) |
|  | FRAAL2940 | 284 | Radical SAM domain protein |
|  | FRAAL2941 | 105 | Hypothetical protein |
|  | FRAAL2942 | 288 | PHP -like protein |
|  | FRAAL2943 | 270 | Putative S-adenosyl-L-methionine-dependent methyltransferase, MT_19 |
|  | FRAAL2944 | 122 | Hypothetical protein |
|  | FRAAL2945 | 147 | Hypothetical protein |
|  | FRAAL2946 | 91 | Hypothetical protein |
|  | FRAAL2947 | 375 | Hypothetical protein |
|  | FRAAL2948 | 205 | Resolvase/integrase-like protein, Res |
|  | FRAAL2949 | 61 | Hypothetical protein |
|  | FRAAL2950 | 86 | Hypothetical protein |
|  | FRAAL2951 | 57 | Hypothetical protein |
|  | FRAAL2952 | 82 | Hypothetical protein |
|  | FRAAL2953 | 121 | Response regulator receiver protein, Res_Reg |
| **Faln5456** | FRAAL 5456 | 479 | Integrase Int |
|  | FRAAL 5455 | 67 | Hypothetical protein |
|  | FRAAL 5454 | 99 | Hypothetical protein |
|  | FRAAL 5453 | 67 | Hypothetical protein (lambda repressor-like DNA-binding domains) |
|  | FRAAL 5452 | 63 | Excisionase Xis |
|  | FRAAL 5451 | 558 | Replication initiator protein RepSA |
|  | FRAAL 5450 | 522 | Transfer protein TraSA cell division FtsK/SpoIIIE |
|  | FRAAL 5449 | 72 | Hypothetical protein |
|  | FRAAL 5448 | 134 | Plasmid replication, integration and excision activator Pra |
|  | FRAAL 5447 | 139 | Transcriptional regulator LacI family |
|  | FRAAL 5446 | 150 | Putative modular polyketide synthase, PKS |
|  | FRAAL 5445 | 278 | Putative S-adenosyl-L-methionine-dependent methyltransferase, MTase_19 |
|  | FRAAL 5444 | 76 | Hypothetical protein |
|  | FRAAL 5443 | 328 | Transcriptional regulator, XRE family |
|  | FRAAL 5442 | 388 | Hypothetical protein |
|  | FRAAL 5441 | 20 | Hypothetical protein |
|  | FRAAL 5440 | 260 | CobQ/CobB/MinD/ParA nucleotide binding domain, CbiA |
|  | FRAAL 5439 | 37 | Hypothetical protein |
| **Fcci1033** | Francci3_1033 | 489 | Integrase, Int |
|  | Francci3_1032 | 99 | Hypothetical protein |
|  | Francci3_1031 | 64 | Excisionase Xis |
|  | Francci3_1030 | 572 | Replication initiator protein RepSA |
|  | Francci3_1029 | 74 | Hypothetical protein |
|  | Francci3_1028 | 519 | Transfer protein TraSA cell division FtsK/SpoIIIE |
|  | Francci3_1027 | 95 | Hypothetical protein |
|  | Francci3_1026 | 134 | Plasmid replication, integration and excision activator, Pra |
|  | Francci3_1025 | 126 | Transcriptional regulator XRE family |
|  | Francci3_1024 | 129 | Hypothetical protein |
|  | Francci3_1023 | 104 | Hypothetical protein |
|  | Francci3_1022 | 441 | Transcriptional regulator XRE family |
|  | Francci3_1021 | 224 | 2’-5’ RNA ligase, LigT |
|  | Francci3_1020 | 197 | Phosphoribosyl glycinamide formyltransferase, PurN |
|  | Francci3_1019 | 116 | Hypothetical protein |
|  | Francci3_1018 | 387 | ISRSO5-transposase |
|  | Francci3_1017 | 139 | Hypothetical protein |
| **Fcci3390** | Francci3_3388 | 508 | Phage Integrase, Int |
|  | Francci3_3389 | 125 | Hypothetical protein |
|  | Francci3_3390 | 380 | Integrase, Int |
|  | Francci3_3391 | 236 | Hypothetical protein |
|  | Francci3_3392 | 479 | Twin-arginine translocation pathway signal |
|  | Francci3_3393 | 366 | Hypothetical protein |
|  | Francci3_3394 | 160 | Hypothetical protein |
|  | Francci3_3395 | 729 | WD-40 repeat-containing serine/threonin protein kinase, P-kin |
|  | Francci3_3396 | 176 | Hypothetical protein |
|  | Francci3_3397 | 111 | Putative regulatory protein, Pra |
|  | Francci3_3398 | 560 | Transfer protein TraSA cell division FtsK/SpoIIIE |
|  | Francci3_3399 | 476 | Putative replication initiation protein, RepSA |
|  | Francci3_3400 | 67 | Excisionase DNA-binding, Xis |
| **Fcci4274** | Francci3_4274 | 407 | Phage integrase Int |
|  | Francci3_4273 | 67 | Excisionase DNA-binding, Xis |
|  | Francci3_4272 | 506 | Replication initiator protein RepSA |
|  | Francci3_4271 | 530 | Transfer protein TraSA cell division FtsK/SpoIIIE |
|  | Francci3_4270 | 130 | Plasmid replication, integration and excision activator, Pra |
|  | Francci3_4269 | 254 | Transcriptional regulator, family GntR, KorSA |
| **FeuI0027** | FraEuI1c_0027 | 397 | Integrase family protein, Int |
|  | FraEuI1c_0026 | 87 | Hypothetical protein |
|  | FraEuI1c_0025 | 102 | Hypothetical protein |
|  | FraEuI1c_0024 | 119 | Hypothetical protein |
|  | FraEuI1c_0023 | 63 | DNA binding domain protein, excisionase family, Xis |
|  | FraEuI1c_0022 | 555 | Replication initiator protein, RepSA |
|  | FraEuI1c_0021 | 565 | Transfer protein TraSA cell division FtsK/SpoIIIE |
|  | FraEuI1c_0020 | 115 | Hypothetical protein |
|  | FraEuI1c_0019 | 137 | Plasmid replication, integration and excision activator Pra |
|  | FraEuI1c_0018 | 129 | Transcriptional regulator, XRE family |
|  | FraEuI1c_0017 | 168 | Hypothetical protein |
|  | FraEuI1c_0016 | 455 | Helix-turn-helix domain protein, HTH |
|  | FraEuI1c_0015 | 224 | Phenazine biosynthesis PhzC/PhzF protein |
|  | FraEuI1c_0014 | 289 | Nucleotide-binding protein, Nbp |
|  | FraEuI1c_0013 | 58 | Hypothetical protein |
| **FeuI6863** | FraEuI1c_6863 | 413 | Integrase family protein, Int -xerC |
|  | FraEuI1c_6862 | 114 | DNA binding domain protein, excisionase family, Xis |
|  | FraEuI1c_6861 | 510 | Replication initiator protein, RepSA |
|  | FraEuI1c_6860 | 102 | Hypothetical protein |
|  | FraEuI1c_6859 | 141 | Single-strand binding protein, Ssb |
|  | FraEuI1c_6858 | 52 | Zinc finger, C2H2-like protein |
|  | FraEuI1c_6857 | 143 | Hypothetical protein (DUF3307 domain) |
|  | FraEuI1c_6856 | 56 | Hypothetical protein |
|  | FraEuI1c_6855 | 60 | Hypothetical protein |
|  | FraEuI1c_6854 | 770 | Cell division protein FtsK/SpoIIIE, TraSA  AAA-like domain |
|  | FraEuI1c_6853 | 387 | Translation initiation factor IF-2 |
|  | FraEuI1c_6852 | 170 | Hypothetical protein |
|  | FraEuI1c_6851 | 197 | Transcription factor WhiB |
|  | FraEuI1c_6850 | 202 | Helix-turn-helix XRE-family like proteins |
|  | FraEuI1c_6849 | 122 | CutA1 divalent ion tolerance protein |
|  | FraEuI1c_6848 | 58 | Hypothetical protein |
|  | FraEuI1c_6847 | 67 | Hypothetical protein |
| **Feun0941** | FrEUN1fDRAFT_0941 | 476 | Integrase family protein, Int |
|  | FrEUN1fDRAFT_0940 | 69 | Hypothetical protein |
|  | FrEUN1fDRAFT_0939 | 100 | Hypothetical protein |
|  | FrEUN1fDRAFT_0938 | 58 | Hypothetical protein |
|  | FrEUN1fDRAFT_0937 | 71 | DNA binding domain protein, excisionase family, Xis |
|  | FrEUN1fDRAFT_0936 | 544 | Putative replication initiation protein, RepSA |
|  | FrEUN1fDRAFT_0935 | 553 | Transfer protein TraSA cell division FtsK/SpoIIIE |
|  | FrEUN1fDRAFT_0934 | 97 | Hypothetical protein |
|  | FrEUN1fDRAFT_0933 | 136 | Plasmid replication, integration and excision activator Pra |
|  | FrEUN1fDRAFT_0932 | 156 | Transcriptional regulator LacI family |
|  | FrEUN1fDRAFT_0931 | 159 | Hypothetical protein |
|  | FrEUN1fDRAFT_0930 | 322 | Transcriptional regulator, XRE family |
|  | FrEUN1fDRAFT_0929 | 398 | Hypothetical protein |
|  | FrEUN1fDRAFT_0928 | 75 | Hypothetical protein |
|  | FrEUN1fDRAFT_0927 | 952 | Putative signal transduction protein with Nacht domain, NTPase |
| **Feun3577** | FrEUN1fDRAFT_3577 | 422 | Integrase family protein, Int |
|  | FrEUN1fDRAFT_3576 | 99 | Hypothetical protein |
|  | FrEUN1fDRAFT_3575 | 62 | DNA binding domain protein, excisionase family, Xis |
|  | FrEUN1fDRAFT_3574 | 479 | Putative replication initiation protein, RepSA |
|  | FrEUN1fDRAFT_3573 | 523 | Transfer protein TraSA cell division FtsK/SpoIIIE |
|  | FrEUN1fDRAFT_3572 | 99 | Hypothetical protein |
|  | FrEUN1fDRAFT_3571 | 134 | Plasmid replication, integration and excision activator Pra |
|  | FrEUN1fDRAFT_3570 | 171 | Transcriptional regulator, XRE family |
|  | FrEUN1fDRAFT_3569 | 162 | Hypothetical protein |
|  | FrEUN1fDRAFT_3568 | 89 | Hypothetical protein |
|  | FrEUN1fDRAFT_3567 | 282 | Transcriptional regulator, XRE family |
|  | FrEUN1fDRAFT_3566 | 156 | MutT (NUDIX domain) |
|  | FrEUN1fDRAFT_3565 | 182 | Metal dependent phosphohydrolase (HD domain) |
|  | FrEUN1fDRAFT_3564 | 300 | Protein of unknown function DUF262 |
|  | FrEUN1fDRAFT_3563 | 312 | Hypothetical protein |
|  | FrEUN1fDRAFT_3562 | 947 | Hypothetical protein |
| **Fdat2245** | FsymDgDRAFT_2245 | 478 | Integrase, Int |
|  | FsymDgDRAFT_2244 | 89 | Phage transcriptional regulator, AlpA |
|  | FsymDgDRAFT_2243 | 535 | Conserved hypothetical protein |
|  | FsymDgDRAFT_2242 | 608 | DNA polymerase family A, Rep |
|  | FsymDgDRAFT_2241 | 108 | Conserved hypothetical protein |
|  | FsymDgDRAFT_2240 | 300 | Bifunctional DNA primase/polymerase, Prim-Pol |
|  | FsymDgDRAFT_2239 | 250 | DNA polymerase III beta subunit, central domain (DNA_pol3_beta_2) |
|  | FsymDgDRAFT_2238 | 162 | Single-strand DNA-binding protein, Ssb |
|  | FsymDgDRAFT_2237 | 144 | Hypothetical protein |
|  | FsymDgDRAFT_2236 | 61 | Hypothetical protein |
|  | FsymDgDRAFT_2235 | 60 | Hypothetical protein |
|  | FsymDgDRAFT_2234 | 739 | Transfer protein TraSA cell division FtsK/SpoIIIE |
|  | FsymDgDRAFT_2233 | 78 | Hypothetical protein |
|  | FsymDgDRAFT_2232 | 260 | Hypothetical protein |
|  | FsymDgDRAFT_2231 | 293 | Hypothetical protein |
|  | FsymDgDRAFT_2230 | 254 | Transcriptional regulator, XRE family |
|  | FsymDgDRAFT_2229 | 157 | MutT (NUDIX domain) |
|  | FsymDgDRAFT_2228 | 190 | Metal dependent phosphohydrolase (HD sub domain) |
|  | FsymDgDRAFT_2227 | 150 | Transcriptional regulator,GntR family |
| **Fdat4298** | FsymDgDRAFT_4298 | 446 | Integrase family protein, Int |
|  | FsymDgDRAFT_4297 | 61 | DNA binding domain protein, excisionase family, Xis |
|  | FsymDgDRAFT_4296 | 506 | Putative replication initiation protein, RepSA |
|  | FsymDgDRAFT_4295 | 526 | Transfer protein TraSA cell division FtsK/SpoIIIE |
|  | FsymDgDRAFT_4294 | 80 | Hypothetical protein |
|  | FsymDgDRAFT_4293 | 54 | Hypothetical protein |
|  | FsymDgDRAFT_4292 | 48 | Hypothetical protein |
|  | FsymDgDRAFT_4291 | 135 | Hypothetical protein |
|  | FsymDgDRAFT_4290 | 258 | Transcriptional regulator, GntR family, KorSA |
