# Supplementary material for: Uncovering the Prevalence and Diversity of Integrating Conjugative Elements in Actinobacteria
Source: PLoS One. 2011 Nov 16;6(11):e27846. doi: 10.1371/journal.pone.0027846 (PMC3218068; doi:10.1371/journal.pone.0027846)
Supplement: Table S9 — Primers used in this study. (DOC) [file pone.0027846.s013.doc]

Table S9: Sets of primers (5’-3’) used for PCRs.

| **AICE** | **Outer primer pairs** | **Inner primer pairs** | **Expected fragment**  **size (bp)** |
| --- | --- | --- | --- |
| Fean1457 | 1457Fw1 CGGTTGAAGGACTGGGCGACG  1457Rv1 CTTGGTCTTGGCGTGGCGTTC | 1457Fw2 GCGGCTCTGGGGTCAGCG  1457Rv2 CCGTCGCCGTACTTCGCCGT | 506 |
| Fean5323 | 5323Fw1 TCACGACGAAGATCGGGACT  5323Rv1 ACCGAAACCCTCCTTGGTTG | 5323Fw2 CGGACAACCTGCGCCGGTTC  5323Rv2 GCGCCAGAGGGTCATCGGCAA | 613 |
| Fean5518 | 5518Fw1 TCCAGCTCACCATGAACACC  5518Rv1 GACCCCGACAGACTCTCCTT | 5518Fw2 TGATGCCGGAGACCCAGCGC  5518Rv2 CACGCCGCCAGGTCACCGAC | 519 |
| Fean5534 | 5534Fw1 ATGGCAAGCCTGCTGCTCGC  5534Rv1 AGCCGGCAGACATGCACAGACG | 5534Fw2 CACTCGCAGATCAGCCTGAC  5534Rv2 CGGACGGAGCTCACAAGGTC | 510 |
| Fean5518 - Fean5534 | 5518Fw1 TCCAGCTCACCATGAACACC  5534Rv3 TGATCCCGGACGTCGCTAG | 5518Fw2 TGATGCCGGAGACCCAGCGC  5534Rv4 CCCACGATGCGGAGAGCAGC | 513 |
| Fean6303 | 6303Fw1 ATCCAGTTACCCTTGCGGCG  6303Rv1 ACCCCGACAGACTCTCCTTCA | 6303Fw2 TGCCCTCCTTCGTCATCCAT  6303Rv2 GATCACACGCCGCCAGGT | 538 |
| Fean6303 | 6303Fw1 ATCCAGTTACCCTTGCGGCG  6336Rv1 CGCTGCCAGGTGGGACAAG | 6303Fw2 TGCCCTCCTTCGTCATCCAT  6336Rv2 ATACCATCGCCGAAGAGCAGC | 357 |
| Faln1739 | 1739Fw1 CGCTCGCCAGTCCGTTGTGGAG  1739Rv1 GCACTCCACGATCCAGCTCACC | 1739Fw2 GTTCGGGTCGGGCCAGGTT  1739Rv2 CACCCACGTGATGCCCGAAAC | 540 |
| Faln2929 | 2929Fw1 ACGCAGCACCTCACACAGCT  2929Rv1 CTTGCGGGACCGTGACCACG | 2929Fw2 ACGGACCAACGTGAGACACA  2929Rv2 CGGTCTTCCGGCAGATGACC | 430 |
| Faln5456 | 5456Fw1 ACTACGTGCCCTCTCCTCGC  5456Rv1 GGAACCCGACTCCGCGTAGATC | 5456Fw2 CTGCCGCGACGCTTCCGCT  5456Rv2 CGGCAACGGGTGTGGGGCTT | 500 |
| Fcci0407 | 0407Fw1 TGTCGGGTCGGCCACGACGA  0407Rv1 GTGAGGCAGACGAGGGTGCCG | 0407Fw2 GTATGCTCCCGGATCTCAGC  0407Rv2 CGCAGTACCGTTCGCCAA | 340 |
| Fcci1033 | 1033Fw1CTGACGGACACCAGCAGAC  1033Rv1 GGTCATGGAGATCATGGGGC | 1033Fw2 CGTCGGCGGAAGATTCAGAT  1033Rv2 ACTCGCAGATCAGCCTGACC | 487 |
| Fcci3390 | 3390Fw1 CGACACCGAGATCTAGACTG  3390Rv1 ATCTACAAATGGCGCACCAC | 3390Fw2 CCTCATACTAGCCTGATCGTC  3390Rv2 CTGACTACCTGCGGTAACCA | 530 |
| Fcci3390_2 | 3390Fw3 TGAGAACGGGCGACAGTATC  3390Rv1 ATCTACAAATGGCGCACCAC | 3390Fw4 GGCGGATGTTCTGGACTAGC  3390Rv2 CTGACTACCTGCGGTAACCA | 609 |
| Fcci4274 | 4274Fw1 GCACGCCACGCCGAACAG  4274Rv1 GGGGTGCCTCGGATCACAGTG | 4274Fw2 CCTCCCTCCGGTCGGCAC  4274Rv2 CCTGTGCGTCGCTGCTGGTG | 470 |
